# Bioinspired Heat Exchangers: A Multi-Scale Review of Thermo-Hydraulic Performance Enhancement

**DOI:** 10.3390/biomimetics11010076

**Published:** 2026-01-16

**Authors:** Hyunsik Yang, Jinhyun Pi, Soyoon Park, Wongyu Bae

**Affiliations:** Department of Electrical Engineering, Soongsil University, Seoul 06978, Republic of Korea; didgustlr02@soongsil.ac.kr (H.Y.); seoulpi31@soongsil.ac.kr (J.P.); syee20221239@soongsil.ac.kr (S.P.)

**Keywords:** biomimetics, heat exchangers, thermo-hydraulic performance, multi-scale, pressure drop, flow network

## Abstract

Heat exchangers are central to energy and process industries, yet performance is bounded by the trade-off between higher heat transfer and greater pressure drop. This review targets indirect-type heat exchangers and organizes bioinspired strategies through a multi-scale lens of surface, texture, and network scales. It provides a structured comparison of their thermo-hydraulic behaviors and evaluation methods. At the surface scale, control of wettability and liquid-infused interfaces suppresses icing and fouling and stabilizes condensation. At the texture scale, microstructures inspired by shark skin and fish scales regulate near-wall vortices to balance drag reduction with heat-transfer enhancement. At the network scale, branched and bicontinuous pathways inspired by leaf veins, lung architectures, and triply periodic minimal surfaces promote uniform distribution and mixing, improving overall performance. The survey highlights practical needs for manufacturing readiness, durability, scale-up, and validation across operating ranges. By emphasizing analysis across scales rather than reliance on a single metric, the review distills design principles and selection guidelines for next-generation bioinspired heat exchangers.

## 1. Introduction

Heat exchangers transfer, distribute, or exchange heat between streams separated by a solid wall. They regulate fluid temperatures through cooling or thermal spreading to an appropriate level. They critically determine energy efficiency and process stability across virtually all real-world industries, including the chemical industry, refining, power generation, HVAC, food processing, and electronics.

Moreover, heat exchangers are expanding their applications beyond conventional industrial processes to encompass thermal management in electronics and data centers, energy storage systems, and next-generation power and hydrogen infrastructures, directly contributing to improved overall system efficiency. In particular, as the share of clean energy increases, driven by global carbon-neutrality goals, hydrogen-based energy systems—especially those utilizing liquid hydrogen—and thermal energy storage technologies have emerged as key solutions for mitigating power-grid instability caused by the intermittency of renewable sources. Since heat exchangers serve as core components in these systems, enhancing their performance and efficiency has become a significant and timely challenge in the field of modern energy management.

The fundamental challenge in enhancing heat exchanger performance lies in achieving an optimal balance between heat transfer efficiency and pressure drop. Heat exchangers are designed to intentionally generate vortices to promote fluid mixing and extend residence time, thereby improving thermal performance, which can be quantified through metrics such as the heat transfer rate or condensation heat flux. Controlled vortex formation can strengthen these parameters; however, excessive turbulence increases flow resistance, weakening the driving pressure of the fluid. This ultimately requires greater pumping power to maintain flow, leading to energy inefficiency. Typically, hydraulic performance is quantified by pressure drop or frictional drag. These metrics represent the energy required to move the fluid. A higher pressure drop implies greater pumping power requirements and higher operational energy consumption.

Because hydraulic performance and heat transfer performance exhibit an inherent trade-off relationship, a performance evaluation criterion (PEC) is commonly employed to comprehensively assess both aspects. The PEC serves as an integrated metric that simultaneously evaluates the benefits of enhanced heat transfer and the energy losses associated with pressure drop. Therefore, maximizing the PEC through well-balanced heat exchanger geometry design and optimized operating conditions is essential for achieving superior overall performance and energy efficiency.

In addition to balancing thermal and hydraulic characteristics, the methods for enhancing heat exchanger performance vary widely depending on the type, structural configuration, and industrial application. Heat exchangers can take several forms, such as shell-and-tube, plate–fin, printed circuit, and microchannel designs. Each type has distinct characteristics, advantages, and limitations derived from its geometry. These structural differences serve as key criteria for selection and evaluation during process design, and accordingly, the strategies for performance enhancement must be tailored to the specific configuration of each heat exchanger type.

For example, a plate heat exchanger (PHE) consists of a series of thin metal plates stacked together to form narrow flow passages, where surface patterns are engineered to guide fluid motion and facilitate efficient heat transfer between the plates. PHEs are valued for their compactness, ease of installation, and high heat transfer performance relative to volume. However, the narrow flow channels make them susceptible to fouling and clogging, which can lead to significant pressure losses. To address these challenges, surface engineering techniques—such as anti-fouling and anti-icing surface modifications—can be applied to enhance performance. Moreover, because the surface texture plays a decisive role in directing fluid flow, the design and optimization of surface patterns have become critical research objectives for improving the efficiency of plate heat exchangers.

A printed circuit heat exchanger (PCHE) is fabricated by micromachining extremely fine channels into metal plates, allowing the device to operate under high-pressure and high-temperature conditions while maintaining a compact form factor with a large heat transfer area per unit volume. However, PCHEs present several challenges, including complex manufacturing and inspection processes and high production costs. Furthermore, their performance can deteriorate significantly if the flow is not evenly distributed through the inlet and outlet manifolds. Therefore, for PCHEs in particular, the design of an optimized flow distribution network is crucial to achieving uniform flow and maintaining consistent thermal performance across the entire heat exchanger.

A microchannel heat exchanger (MCHE) features a flat-plate structure composed of numerous narrow parallel channels and manifold networks, which maximizes surface contact area per volume and makes it highly effective for phase-change heat transfer processes such as evaporation and condensation. In addition, MCHEs offer advantages in lightweight design and compactness. However, due to their microscale channel dimensions, they are highly sensitive to particulate contamination and prone to blockage, flow maldistribution, and relatively large pressure losses. Moreover, manufacturing inconsistencies can lead to significant performance variations among individual units, posing a continuing challenge for reliable large-scale implementation.

To address the key factors that cause performance degradation in heat exchangers and to further enhance their efficiency, nature-inspired technologies have recently gained increasing attention. Over millions of years of evolution, many organisms have developed the ability to maximize heat and mass transfer using minimal energy, as well as to reduce friction and prevent fouling in fluid environments. These natural characteristics provide valuable insights for tackling the fundamental challenges of heat exchanger performance enhancement.

In this study, recent advances in bioinspired heat exchanger technologies are systematically reviewed based on representative research cases, offering an organized overview of the state-of-the-art trends in this field. Section 2 describes the scope and classification framework of the reviewed literature, while Section 3 presents detailed research examples categorized by biomimetic scale and mechanism. Finally, Section 4 synthesizes the reviewed studies to discuss emerging trends, limitations, and future research directions in bioinspired heat exchanger design.

## 2. Methodology and Classification

### 2.1. Scope of the Review

The primary objective of this study is to explore bioinspired technologies applied to the performance enhancement of heat exchangers, which are devices designed to facilitate thermal transfer and exchange using fluids. However, heat exchangers exhibit a wide variety of purposes, configurations, and applications, and the concept of “heat exchange” itself can be interpreted differently across industrial contexts. Therefore, this section establishes the inclusion and exclusion criteria used in this review to define the scope of representative heat exchanger technologies, ensuring a clear and systematic framework for subsequent classification and analysis.

In this review, the term heat exchanger specifically refers to thermal management devices that facilitate heat transfer, diffusion, and exchange using fluids. Accordingly, the scope excludes systems including heat sinks designed solely for dissipating heat from electronic components, heat pumps that rely on fluid circulation and refrigeration or heating cycles for space conditioning, and latent heat storage systems that utilize phase change materials (PCMs) to maintain stable temperatures over extended periods, such as in building applications.

Furthermore, this review focuses exclusively on indirect-type heat exchangers, in which two fluids are separated by a solid wall and do not come into direct contact. Therefore, systems that involve direct mixing between fluids or technologies that manage the thermal effects arising from fluid mixing are excluded from the scope of this study.

However, certain technologies—such as latent heat storage systems, vapor chambers, heat sinks, and metal surface modification techniques—can be closely associated with heat exchangers, as they are either already applied in heat exchanger systems or possess high potential for practical integration. For instance, Li et al. (2025) [1] applied a lotus-leaf-inspired superhydrophobic coating to the fins of a heat pump to mitigate efficiency losses caused by condensation and frost formation—a mechanism directly analogous to that of frost prevention in heat exchangers. Therefore, even when a study does not strictly pertain to a heat exchanger in the narrow sense, technologies with strong applicability or transfer potential to heat exchangers are additionally reviewed. In such cases, the relevance and applicability to heat exchanger systems are explicitly discussed from a rational and engineering perspective.

The literature search for this review was primarily conducted using major academic publisher platforms, including MDPI, ScienceDirect, Wiley Online Library, SpringerLink, and Scopus. In addition, forward and backward citation tracing was performed using related review papers, as well as the references and citations of key studies, to expand the scope of the relevant literature. To ensure broader coverage and diversity of sources, Google Scholar was also utilized as a supplementary search channel. The literature search was conducted from 15 August to 9 October 2025, focusing mainly on studies published after 2010 while prioritizing works published after 2020 to accurately capture the latest trends in biomimetic heat exchanger research.

Generative AI tools were used only for the creation of some illustrative schematic images to improve conceptual clarity; no experimental or simulation data were generated or modified using AI.

### 2.2. Classification

**Figure 1:**
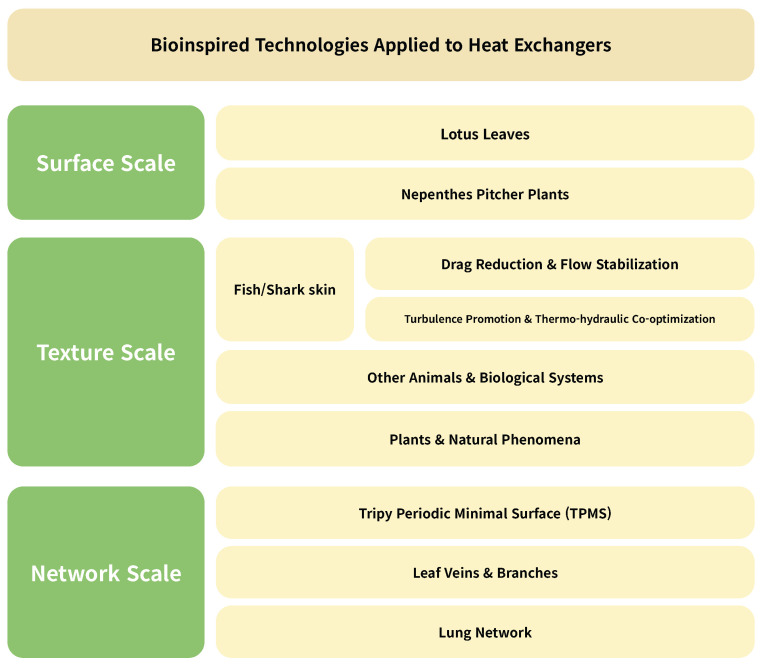
Hierarchical classification of bioinspired technologies applied to heat exchangers. Bioinspired technologies for enhancing heat exchanger performance are categorized into three hierarchical scales: Surface Scale (focusing on micro/nanoscale surface phenomena like Lotus Leaves and Nepenthes Pitcher Plants), Texture Scale (focusing on surface patterns and protrusions to control fluid motion, including Fish/Shark Skin, Other Animals & Biological Systems, and Plants & Natural Phenomena), and Network Scale (focusing on macroscopic flow optimization, including Triply Periodic Minimal Surface, Leaf Veins & Branches, and Lung Network). Green boxes indicate the hierarchical scales (surface/texture/network), whereas beige boxes denote representative bioinspired motifs/examples within each scale.

As shown in Figure 1, bioinspired technologies for enhancing heat exchanger performance are categorized into three hierarchical scales: Surface, Texture, and Network. The surface scale, representing the microscale to nanoscale level, focuses on techniques that mimic natural surface phenomena to improve the thermal efficiency of heat exchangers. This category primarily includes methods that modify surface properties to enhance slipperiness, suppress frost formation or icing, and in cases involving condensation, prevent liquid film formation that can hinder effective heat transfer.

The texture scale represents an intermediate level of biomimetic design, focusing on techniques that enhance heat exchanger performance by controlling fluid motion through surface patterns or protrusions inspired by natural textures. While it shares with the surface scale the concept of modifying the solid wall surface, the texture scale differs in its primary objective—to regulate fluid flow in a manner that reduces friction and pressure drop while simultaneously generating beneficial vortices that promote heat transfer. This approach enables the simultaneous achievement of lower pumping power requirements and higher thermal efficiency, both of which are critical for the effective operation of heat exchangers.

The network scale shares similar objectives with the texture scale; however, instead of focusing on microscopic surface alterations such as patterning, it focuses on heat exchanger performance from a macroscopic perspective by optimizing the fluid pathways themselves, thereby preventing unnecessary increases in pumping power. Representative strategies include topological designs that induce appropriate vortices through three-dimensional geometries of flow conduits (e.g., cylindrical channel architectures) or the placement of flow obstacles along the path, as well as layout optimization of pipe networks and other routing schemes that govern how the working fluid is distributed and mixed throughout the exchanger.

However, in certain heat exchanger configurations, the distinction between the texture scale and the network scale becomes less clear. This ambiguity is particularly evident in compact designs such as MCHE and PCHE, where the flow passages are extremely small or planar—in such cases, the surface protrusions themselves effectively form the fluid pathways. Consequently, differentiating between surface-driven and flow-path-driven mechanisms can be challenging. To address this issue, the present review distinguishes each study based on whether its primary performance indicators and analyses focus on surface-level flow behavior or on flow-field optimization beyond the surface. For example, the texture scale focuses on optimizing the near-wall boundary layer, whereas the network scale aims to optimize the flow behavior across the entire cross section of the channel.

### 2.3. Performance Evaluation and Validation of Numerical Analysis

This review evaluates each study by focusing on the performance bottlenecks and operating windows beyond which the improvement diminishes. It emphasizes that, even for the same biomimetic geometry, superiority can be reversed depending on the selection of the baseline (comparative reference geometry), and it critically examines the risk of overinterpretation when improvement figures presented under different metrics and baselines are compared across studies.

In particular, it is necessary to exercise caution regarding this tendency in studies that conduct only numerical analyses. Some of the studies reviewed in this paper mention only numerical analysis results without experimental outcomes. These results should be interpreted as evidence of trends and relative superiority under identical conditions rather than as absolute performance conclusions. In practical applications, real-world factors such as clogging in compact structures, flow non-uniformity, and manufacturing deviations may reverse the performance ranking.

Therefore, to mitigate these issues, the validation frameworks were summarized for some topics in which many studies are based on numerical analyses. Based on the validation information reported in each study, this review assessed whether the numerical analyses represent practical heat-exchanger conditions. Accordingly, this paper classifies computational fluid dynamics (CFD)-based claims reported in individual studies as more and less robust, guiding readers to interpret the literature not merely by performance figures, but by design feasibility considering fabrication and operation.

## 3. Bioinspired Technologies Applied to Heat Exchangers

### 3.1. Surface Scale

Low-temperature heat exchangers often suffer from a chronic efficiency degradation caused by liquid film formation or frost accumulation. To prevent this, researchers have developed surface modification technologies that allow condensed droplets to slide off naturally or suppress frost formation, such as by introducing superhydrophobic coatings or slippery interfaces. These water-repellent mechanisms are commonly observed in nature, particularly on plant surfaces.

For example, Wu et al. (2023) [2] designed a double-gradient microgrooved structure inspired by cactus spines and spider silk. A layer of copper oxide nanostructures was formed on a copper substrate, followed by fluorosilane treatment to achieve superhydrophobicity with a contact angle of 157°. Condensation experiments revealed that the droplet driving force increased by up to 37.9% compared with single-wave grooves, and the condensation heat flux improved by 18–22% relative to a single-structure surface and by 27–31% compared to a flat surface.

At the surface scale, such bioinspired structural modifications enable superhydrophobicity on heat exchanger surfaces without altering the overall geometry, thereby preventing frost formation and liquid film accumulation. Consequently, these approaches effectively eliminate potential causes of performance degradation, providing a simple yet powerful route to enhance the thermal efficiency of heat exchangers.

#### 3.1.1. Lotus Leaves

**Figure 2:**
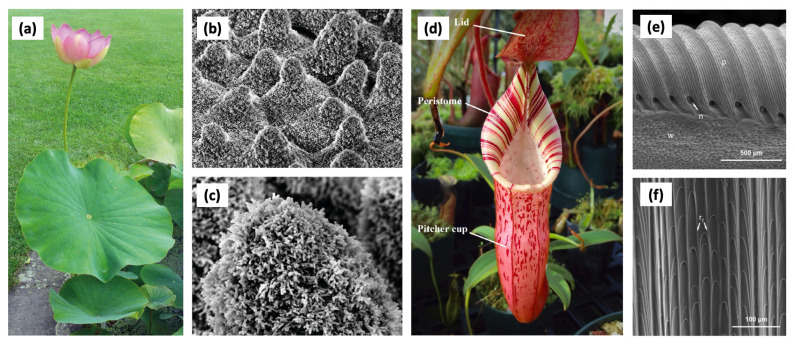
Bioinspired surfaces for enhanced thermal efficiency (Surface Scale). (**a**) A lotus plant exhibiting superhydrophobic leaves. (**b**) Scanning electron microscope (SEM) image showing the microstructures and (**c**) nanorods on the lotus leaf surface, which synergistically promote superhydrophobicity and droplet shedding. (**d**) An image of a Nepenthes pitcher plant, highlighting the lid, peristome, and pitcher cup, which possesses a super slippery surface. (**e**) SEM image showing the microstructures (p: peristome, n: nectary, w: wax layer) and (**f**) nanostructures on the peristome surface, enabling a continuous and low-friction liquid interface ((**a**–**c**) adapted from [3], (**d**–**f**) adapted from [4]).

The lotus leaf is one of the most representative natural motifs exhibiting superhydrophobicity. As shown in Figure 2a–c, its surface achieves a large contact angle through the synergistic combination of micro/nanostructures and waxy secretions, allowing water droplets to easily roll off the surface. This mechanism not only removes contaminants from the surface but also helps prevent liquid film formation and frost accumulation. This section introduces research efforts that have replicated the superhydrophobic surface of lotus leaves to achieve frost prevention and thermal efficiency enhancement in heat exchangers.

Shen et al. (2025) [5] proposed an innovative biomimetic vapor chamber design that emulates the efficient moisture management and structural characteristics found in nature to enhance thermal performance. Inspired by the superhydrophobic properties of lotus leaves, the condensation surface of the vapor chamber was modified to promote rapid dropwise condensation of condensed liquid. In addition, multiple natural inspirations including superhydrophilic moss, honeycomb lattice architectures, and leaf vein-like capillary networks were incorporated to further optimize liquid transport and structural stability. Experimental results demonstrated that the biomimetic vapor chamber reduced the time to reach steady-state by up to 11.7% and improved the temperature uniformity coefficient by 7.74% compared with conventional designs. A steady-state multiphysics model in COMSOL Multiphysics 6.1 (laminar vapor flow coupled with porous-wick Brinkman transport) showed strong agreement with the experimental temperature field. The simulations further indicated that phase-change (latent-heat) transport dominates the overall heat dissipation, explaining the improved thermal response of the biomimetic design. However, the chemical surface modification method used in this study has durability limitations. Therefore, future research should focus on enhancing surface stability and longevity through laser-based fabrication or chemical vapor deposition techniques.

Li et al. (2025) [1] addressed the efficiency degradation of air-source heat pump (ASHP) aluminum fins caused by frost formation by designing a bioinspired linear micro-pattern on the aluminum surface. The pattern was developed by mimicking the fine ridges of scallop shells and the spacing between protrusions on lotus leaves. This structure effectively restricted droplet spreading and trapped air pockets on the surface, thereby significantly enhancing its hydrophobicity. Furthermore, a polydimethylsiloxane (PDMS) dip-coating was applied to partially fill the microcavities and reduce surface friction, resulting in a smoother droplet motion. The composite surface maintained stable hydrophobicity and low ice adhesion strength even after repeated defrosting tests, demonstrating excellent durability and scalability. These results indicate that the proposed bioinspired PDMS-coated surface represents a promising passive anti-icing technology for optimizing heat exchangers in ASHPs. Future work should focus on parametric optimization of the surface pattern and refinement of the PDMS coating process to further improve performance.

Cheng et al. (2024) [6] simultaneously replicated the multilayer micro/nano-scale hydrophobic surface of lotus leaves and the hollow sandwich (honeycomb interlayer) structure found in nature. A polyurethane foam core was used to construct the sandwich interlayer, while the top surface was engineered with micro-cone arrays and nanorods, achieving superhydrophobicity with a contact angle greater than 150° and a notable delay in droplet freezing. This design effectively combined thermal insulation, anti-icing capability, and mechanical reinforcement within a single architecture. However, the validation was primarily limited to static droplet and constant-temperature indoor tests under a single cooling condition. Therefore, further investigations are required to evaluate performance under fluctuating outdoor environments, as well as long-term durability, wear resistance, large-area manufacturability, and cyclic freeze–thaw behavior when applied to actual heat exchanger surfaces.

Xing et al. (2024) [7] sought to mitigate the frost growth issue on the outdoor heat exchanger of an ASHP by following the trajectory of nature-inspired superhydrophobic surface research. They fabricated a superhydrophobic surface with a contact angle of 156.7°, and comparatively evaluated, through visualization experiments, the frost growth and melting characteristics on finned tubes. As a result, they reported performance gains, with the frost build-up reduced by about 18.9% and the defrosting efficiency enhanced by 21.2%. Furthermore, they analyzed fin pitch, fin surface temperature, and wind speed as parameters affecting frosting characteristics, and organized the data to identify under which operating and geometric conditions the effect becomes larger. However, the evidence presented in this paper is focused on frost growth/defrosting performance and an early-stage mechanistic model. From a practical application perspective, follow-up validation is still needed to link the concurrent effects of fouling, reproducibility under fluctuations in humidity, wind speed, and temperature, and the long-term durability of the superhydrophobic surface.

Lotus leaf-inspired superhydrophobic surface modification applied to heat exchangers can address the problem of increased thermal resistance caused by frost, fouling, and corrosion. However, prior superhydrophobic studies have remained focused on materials and surface-treatment mechanisms, leaving the utility and durability unclear when applied to actual heat-exchanger fins. To resolve this issue, Li et al. (2022) [8] conducted process optimization and durability validation. As a result, they secured superhydrophobicity with a contact angle of 166.9°, and applied this process to a full-size fin-and-tube heat exchanger to demonstrate enhanced hydrophobicity through droplet behavior. In this way, they established a package of a process applicable to heat-exchanger components, wettability, and durability. Further evaluation is needed on how much this package improves actual heat-exchange performance.

Accordingly, lotus-leaf-inspired surface mimicry suppresses droplet spreading and film formation through microscale surface structures, and consequently promotes roll-off and dropwise condensation, reducing liquid-film thermal resistance and frost accumulation. Although the contact angle is predominantly used for performance comparisons, dynamic metrics that critically affect real operation, such as sliding angle, contact angle hysteresis, and the droplet departure period and diameter, were insufficiently reported. The aforementioned studies commonly point out limitations that the structures are vulnerable to wear and contamination, and that the experimental conditions are static. From a practical design perspective, lotus-leaf-inspired surface mimicry is effective under conditions where suppressing initial icing translates into efficiency, such as ASHP fins with a high defrost-cycle burden. However, under conditions with substantial outdoor contamination or wear, and under conditions dominated by thermal-load fluctuations, the simultaneous optimization of durable fabrication processes, thermal resistance, ice resistance, and cleanability is essential.

#### 3.1.2. Nepenthes Pitcher Plants

As shown in Figure 2d–f, Nepenthes pitcher plants possess inner wall microstructures and a thin liquid film that enable insects to slide easily into the trap. Inspired by this natural mechanism, slippery liquid-infused porous surfaces (SLIPS) have been developed by infusing low-viscosity lubricants into nanostructured solid substrates, thereby creating a smooth and defect-free liquid interface. Such surfaces effectively suppress filmwise condensation, while simultaneously reducing thermal resistance, inhibiting frost formation, and preventing surface fouling in heat exchangers. This section introduces research efforts that mimic the slippery surface of Nepenthes pitcher plants to achieve enhanced surface mobility and improved thermal performance in heat exchangers.

Kim et al. (2012) [9] highlighted that conventional lotus-leaf-inspired superhydrophobic surfaces can actually promote ice nucleation and increase ice adhesion strength under high-humidity conditions. To address this issue, they applied the ‘SLIPS’ approach. A nanostructured polypyrrole film was first deposited on an aluminum substrate through electrodeposition, followed by fluorination treatment and infusion of a low-viscosity perfluorinated lubricant, enabling condensed droplets to slide off spontaneously under gravity before freezing. Experimental results showed that the SLIPS-Al surface exhibited an ice adhesion strength as low as 15.6 kPa, which is over ten times lower than that of conventional materials, and effectively suppressed frost accretion even under low-temperature and high-humidity conditions. Moreover, during defrosting cycles, frost and meltwater were immediately removed from the surface, significantly reducing energy consumption. This study demonstrates the strong potential of SLIPS technology as a robust anti-icing material for heat exchanger applications.

In heating, ventilation, air conditioning, and refrigeration systems, managing condensate and frost accumulation on heat exchanger surfaces is crucial for improving energy efficiency, and this challenge can be addressed by mimicking the super-slippery surface mechanism of Nepenthes pitcher plants. To implement this concept, Yu et al. (2014) [10] coated an alumina microstructured layer with a self-assembled monolayer of heptadecafluoro tetrahydrodecyl trichlorosilane, followed by the infusion of a perfluoropolyether lubricant. The resulting slippery surface significantly enhanced droplet mobility by creating a liquid–liquid interface instead of a traditional solid–liquid contact, while simultaneously reducing the droplet contact angle. Moreover, after self-defrosting, the surface exhibited only one-quarter of the water retention observed on a reference surface, confirming its excellent drainage performance. However, oil depletion was observed during testing, indicating the need for future studies to develop strategies that minimize lubricant loss and maintain long-term surface stability.

In vertical condenser tubes, filmwise condensation introduces additional thermal resistance, while hydrophobic and superhydrophobic coatings often suffer from reduced durability and increased interfacial resistance. To overcome these limitations, Abraham et al. (2023) [11] proposed a slippery liquid-infused surface strategy inspired by the lipid gland secretion and self-replenishing mechanism of frog skin as well as the low-friction surface of Nepenthes pitcher plants. In this design, a nonwoven porous tape was attached to the upper section of the condenser tube and impregnated with polytetrafluoroethylene or copper grease, allowing continuous self-replenishment of the slippery layer through cloaking and wedging effects during condensation. Experimental validation showed that the condenser maintained stable dropwise condensation for 10 h per day over three consecutive days without noticeable performance degradation. However, reduced heat transfer performance was observed outside a certain range of subcooling conditions. Therefore, when applying this technique to heat exchangers, further studies are required to optimize material compatibility, contamination resistance, long-term durability, and the replenishment rate of the lubricating layer.

Baba et al. (2021) [12] proposed a strategy to enhance condensation heat transfer by inducing spontaneous droplet jumping, inspired by the hierarchically superhydrophobic surface of Euphorbia myrsinites leaves. They fabricated micro/nanostructured surfaces on silicon wafers by combining reactive ion etching and Bosch processes with thermal dewetting of gold, producing structures that exhibited static contact angles greater than 160° across all samples. When the pillar height was sufficiently large, rebound droplets that reattached after jumping were observed to re-jump repeatedly from pillar tops, leading to the near disappearance of large droplets (>35 μm in diameter) within 10 min. In contrast, surfaces with shorter pillars retained larger droplets. The results clearly demonstrated an improvement in surface refreshment, as indicated by the increased cumulative detached droplet volume. However, further validation is needed to assess the effects of ambient conditions, long-term durability, and contamination resistance.

The air lubricant layer realized by conventional superhydrophobic surfaces has limitations, including low thermal conductivity and instability. To address this, Sirohia et al. (2019) [13] propose an air-independent slippery rough surface that combines the micro/nano texture of the lotus leaf with the slippery principle of the Nepenthes pitcher plant. In their numerical model, under the conditions of Rm = 2 and an intrinsic contact angle ≈ 76, they predict an improvement in the heat transfer coefficient of 191.3% compared with an superhydrophobic fluorinated surface and 407.2% compared with an superhydrophobic surface. It presents a design direction indicating that this surface satisfies both droplet mobility and nucleation without an air layer. However, as the authors note, this model has a limitation in that it cannot directly incorporate dynamic processes such as renucleation over time and sweeping, and thus design and real-environment operation evaluation that consider these effects are needed.

Copper, which is widely used in heat exchangers, has high thermal conductivity, but in real operating environments, it suffers from degraded surface performance due to contamination by dust, oil, and microorganisms and exposure to acids. Ryu et al. (2023) [14] applied the Nepenthes-derived slippery surface concept to a copper substrate, while introducing a silica nanoparticle reinforcing layer to anchor the lubricant more strongly via capillary force. They also propose a strategy in which this layer functions as a physical barrier that prevents external droplets and acids from contacting the substrate. As a result, they showed long-term acid resistance, with the surface maintained even after 4 h of continuous supply, and the cycle at which depletion transition occurs was delayed by about twofold. In addition, after 45 min of dropwise condensation, dust residue was virtually removed and an anti-contamination effect was also reported, and the heat-transfer performance was relatively well maintained even after dust contamination. However, there is a trade-off in that as lubricant viscosity increases, retention improves but droplet mobility decreases, and additional optimization under different operating conditions and long-cycle durability validation are required.

When copper-based components such as heat exchangers are exposed to high-salinity, high-oxygen electrolyte conditions in marine environments, corrosion and biofouling proceed simultaneously, shortening service life, and when a heat exchanger fails, the resulting loss of cooling can even lead to accidents. Yu et al. (2022) [15] present an metal-organic framework-based liquid-infused surface coating strategy to address this issue by combining a liquid-infused surface concept inspired by the slippery surface of Nepenthes with an metal-organic framework porous matrix. By forming a slippery interface in which the lubricant can be anchored and redistributed within the metal-organic framework based porous framework, they achieved a corrosion inhibition efficiency of 99.999%, secured sustained corrosion resistance even after immersion in NaCl solution, and enhanced icephobic performance. However, the intrinsic underwater instability of the metal-organic framework itself and limited long-term durability are identified as limitations. In the future, additional design and validation are needed, such as increasing oil storage capacity with a large-pore metal-organic framework to secure long-term protection, or utilizing it as an additive in commercial coatings.

Accordingly, Nepenthes pitcher-plant-inspired surfaces or SLIPS enhance droplet mobility and hinder ice adhesion and accumulation by forming a lubricant layer and realizing a superhydrophobic structure with ultra-low hysteresis. In the aforementioned papers, convincing performance metrics grounded in experiments, such as frost delay, sliding angle, and ice adhesion strength, were employed. However, because the dominant performance bottlenecks differ, quantitative comparisons were limited. In addition, these studies commonly exhibited issues of constrained operating conditions and long-term stability problems, such as observed lubricant depletion. From a practical design standpoint, this approach is particularly attractive in applications where condensate water must be managed, such as the air side of air conditioning or refrigeration systems. Conversely, in field settings where there is no design to structurally suppress or replenish lubricant loss, where the operating envelope is wide, or where contamination and wear accumulate over the long term, a conservative application or a non-recommendation is reasonable.

#### 3.1.3. Surface Scale Critical Synthesis and Design Guidelines

Surface-scale bioinspired technologies can be interpreted as an approach that secures operational stability by suppressing performance degradation caused by condensation, icing, and contamination, rather than directly increasing the heat transfer coefficient. Both lotus-based superhydrophobic surfaces and Nepenthes-inspired SLIPS enhance droplet mobility and reduce liquid-film/frost accumulation, but their effectiveness is strongly dependent on operating conditions and surface states.

Lotus-inspired superhydrophobic surfaces can induce dropwise condensation and delay initial icing by suppressing droplet spreading via micro-/nanostructures and low surface energy. However, in high-humidity environments, ice adhesion can increase due to enhanced condensation nucleation or moisture entrapment associated with the Cassie–Wenzel transition. Therefore, superhydrophobic surfaces should be evaluated within a performance window defined by humidity, temperature, and condensation intensity.

In addition, the static contact angle does not sufficiently explain real operational performance. From a heat-exchanger perspective, the key evaluation metrics are drainage-dynamics indicators such as sliding angle, contact angle hysteresis, and the critical departure diameter/period. Performance evaluation of surface technologies should be reformulated away from static water repellency and toward dynamic drainage characteristics under condensation/icing conditions.

SLIPS can complement the vulnerable regime of superhydrophobicity by suppressing droplet/frost adhesion under humid, low-temperature conditions through a lubricant layer and ultra-low hysteresis. However, the key bottleneck is long-term stability due to oil depletion, and practical adoption is governed more by lubricant-layer retention/replenishment and maintenance strategies than by initial performance.

Table 1 summarizes the surface-scale bioinspired technologies discussed above as a design decision-making framework. The Works/Fails entries synthesize the valid operating window and failure drivers, which vary with temperature, humidity, contamination, icing, and defrost conditions, and do not indicate an absolute superiority or inferiority of any technology. In addition, based on each referenced work, the key evaluation metrics are presented as recommended performance indicators to improve cross-study comparability and real-operating-condition reproducibility.

In summary, for heat-exchanger applications, surface modification can inversely limit performance through added thermal resistance (coatings/layers), air entrapment, and accumulated wear/contamination. Accordingly, it should be treated as a multi-objective optimization problem that includes thermal resistance, wear resistance, contamination resistance, and repeated-defrost durability, in addition to water repellency and drainage performance. Ultimately, the selection criteria between superhydrophobicity and SLIPS should be organized not by water repellency itself but by a performance-window and life-cycle design perspective that reflects the operating humidity range, icing/defrost conditions, contamination/wear environment, and maintainability.

### 3.2. Texture Scale

Texture-scale bioinspired structures represent an efficient approach to achieving significant thermo-hydraulic performance enhancement by precisely controlling heat and momentum transfer in the microscopic region of the fluid boundary layer without altering the macroscopic geometry. Surface textures found in nature—such as scales, riblets, and micro-protrusions—evolved through long-term adaptation and exhibit two opposite functionalities: they can either promote turbulence to enhance heat transfer or suppress turbulence to reduce frictional drag. Therefore, the key to engineering applications lies in strategically tuning these opposing effects to align with system objectives and identifying the optimal design point that balances the inherent trade-off between heat transfer enhancement and pressure drop minimization.

#### 3.2.1. Fish and Shark Skin

**Figure 3:**
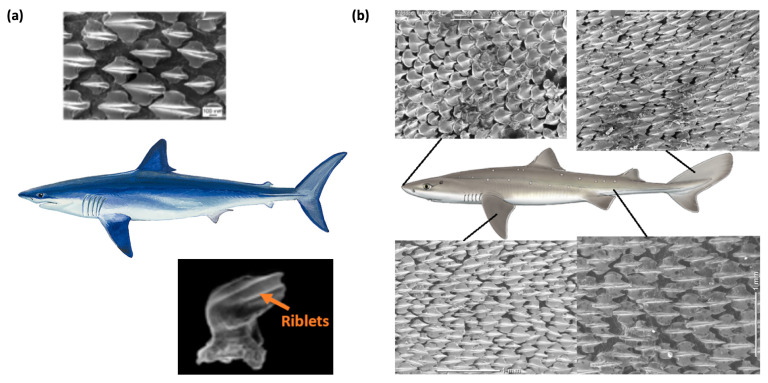
Biological archetype of shark-skin-inspired riblet surfaces. (**a**) Illustration of a fast-swimming shark and magnified views of an individual dermal denticle, highlighting streamwise riblets aligned with the local flow direction [21]. Shark silhouettes are reproduced from an open-source illustration on Wikimedia Commons (CC BY license), and [22] (**b**) scanning electron micrographs of different regions along the shark body, showing variations in denticle packing density and riblet orientation. These multiscale dermal structures act to lift near-wall streamwise vortices and weaken their rotational motion, providing the biological basis for engineered blade-type riblets that achieve up to ≈11.6% drag reduction at optimal non-dimensional spacing and height (s^+^ ≈ 15–20, h^+^ ≈ 8–10) ((**b**) adapted from [23]).

Fish skin provides a quintessential example of a surface that is hydrodynamically optimized for survival in aquatic environments. In particular, the riblet microtextures on shark skin attenuate near-wall turbulent vortices and thereby reduce frictional drag, whereas the scales of typical fish promote vortex generation, enhancing maneuverability and heat dissipation efficiency. This section reinterprets these dual functionalities from an engineering perspective, offering an in-depth analysis of studies that leverage fish-skin-inspired surfaces for two principal objectives: drag reduction and heat transfer enhancement. As shown in Figure 3, shark skin is characterized by streamwise-aligned dermal denticles that create riblet-like microgrooves with locally tuned packing density and orientation, a geometry that modulates near-wall vortical structures and suppresses cross-stream momentum exchange, thereby promoting drag reduction and flow stabilization.

**Drag Reduction and Flow Stabilization**

Dean et al. (2010) [16] synthesized shark-skin riblet studies and noted that peak drag reduction is typically on the order of 10%, while emphasizing an s^+^-based design window in which riblets are generally most beneficial near s^+^ ≈ 15. It also highlights the sensitivity to flow alignment, reporting that riblets can become drag-inducing at yaw angles above β = 30° and recommends a slightly lower spacing for internal/pipe flows (12 < s^+^ < 14). Although this study suggests numerical modelling of turbulent duct flow over virtual riblets using an immersed-boundary approach, reporting up to 3.3% drag reduction and attributing it to vortices remaining above riblet tips, the formation of a low-velocity channel within riblet valleys, and damped vortex translation that suppresses near-wall turbulence events.

As an example of narrowing this viscous-scaled s^+^ design window to riblet-specific optimization, Martin et al. (2016) [17] conducted transient large-eddy simulation (LES) in ANSYS Fluent 16.0 for fully developed turbulent channel flow using a periodic domain to directly compare time-averaged wall drag on riblet versus smooth walls. With the riblet height fixed at h^+^ ≈ 8 and spacing swept over a wide range, the blade-type riblet achieved the maximum drag reduction of 11.6% at s^+^ = 25.3. Beyond reporting the peak value, their flow-structure analysis links performance to quasi-streamwise vortex behavior: overly small spacing increases wetted area and tip–vortex interactions, whereas excessively large spacing allows vortices to penetrate riblet valleys, markedly degrading drag reduction and in some cases even producing net drag increase depending on geometry. Accordingly, this synthesis suggests a practical viscous-scaled design window of s^+^ ≈ 15–20 and h^+^ ≈ 8–10 for robust drag reduction trends across riblet designs.

Beyond conventional two-dimensional linear riblets, researchers have explored three-dimensional geometrical variations to achieve more comprehensive control over near-wall vortical structures. Using direct numerical simulation (DNS), Sasamori et al. (2017) [18] analyzed sinusoidal riblets in fully developed turbulent channel flow at $Re_{\tau }$ = 110, implemented via an immersed-boundary method under homogeneous and no-slip wall conditions; they also checked grid/domain sensitivity and verified key flat-wall statistics (mean velocity and Reynolds shear stress) against prior direct DNS while noting RMS discrepancies due to the small domain. This resulting flow pattern effectively weakens the near-wall vortical structures responsible for turbulent momentum transfer associated with skin-friction drag. In particular, the upward flow induced in the contraction regions pushed vortices away from the wall, resulting in a substantial 9.8% reduction in drag with the net benefit attributed mainly to a strong decrease in the random Reynolds shear stress, consistent with vortex tracking. The study also highlighted the critical importance of the wavelength (λ^+^) as a new design parameter: excessively short wavelengths can induce flow separation within the riblet valley, dramatically increasing pressure drag, thereby offsetting the frictional drag reduction effect. This finding provides a crucial engineering insight—that in three-dimensional riblet design, the complex effects of local geometric variations on the overall flow field must be taken into account.

In contrast to conventional shark-skin riblet studies, Wu et al. (2017) [19] focused on the unique water-trapping mechanism observed on the scaly surfaces of typical fish. The key to this mechanism is to immobilize a near-wall layer using a stable scale microstructure, forming a lubricating layer that acts like a liquid bearing. This lubricating layer reduces the direct friction between the solid surface and the main flow, reducing the wall shear stress and at the same time suppressing the transfer of Reynolds stress caused by turbulence. To quantify this effect, they performed CFD simulations in FLUENT using a 3D simplified scale model RNG k–ε and compared a bionic surface against a smooth reference over low-speed conditions (0.66–0.82 m/s), with specified discretization and convergence criteria. Numerical simulations revealed up to a 3% reduction in drag under specific low-velocity flow conditions, with the reported maximum drag reduction of 3.014% at 0.66 m/s; the drag decomposition further indicated that although pressure drag increases due to the surface relief, the net drag decreases because the viscous component is reduced more strongly. They also checked outlet boundary-condition sensitivity, showing consistent drag-reduction trends despite differences in absolute values. This finding holds significant academic value as it demonstrates an alternative pathway for friction mitigation through liquid-mediated lubrication rather than structural vortex manipulation.

For the commercialization of bioinspired surface structures, the development of practical fabrication techniques is essential. In this regard, Žemaitis et al. (2019) [20] proposed a high-efficiency laser processing method that efficiently manufactures riblets on polymer materials that are difficult to process. By preheating the substrate during laser irradiation, the material’s light absorption increased, improving etching efficiency by 30%. The fabricated surfaces demonstrated a maximum friction reduction of 6% in experimental tests, representing a significant step toward the practical realization of biomimetic structures. However, the low thermal conductivity remains a clear limitation for direct application in heat exchangers, indicating the need for further research into alternative materials or composite fabrication strategies. Table 2 summarizes the maximum drag-reduction rates and the baseline conditions used for comparison, while also capturing the principal trade-offs/boundaries that limit the generality of the reported improvements.

**Table 2:** Summary of maximum drag reduction and key trade-offs/boundaries reported in biomimetic drag-reduction studies.

Biomimetic Target	Maximum Drag Reduction Rate (%)	Comparator (Baseline)	Trade-Off/Boundary	Ref.
Shark Skin Riblets (Blade-type)	11.6	Smooth surface (no riblets)	drag reduction peaks only near the optimized s^+^, h^+^/overly large s^+^ may increase drag	[17]
3D Sinusoidal Riblets	9.8 (±2)	Smooth-wall turbulent channel (no riblets)	pressure drag can rise with higher h^+^/a^+^ or shorter λ^+^/too short λ^+^ can induce separation and negate benefits.	[18]
Ctenopharyngodon Idellus Scale	3.014	Smooth surface	modest drag reduction/higher speed increases near-wall viscous resistance and pressure force	[19]
Shark Skin Riblets	6	Flat, unstructured PTFE	benefit limited to a narrow spacing window; validated only for airflow on PTFE	[20]

The optimal conditions of Martin et al. (2016) [17] and the observation that drag can be increased in excessive s^+^ can be more generally interpreted in terms of regime transition of riblet performance summarized by García-Mayoral et al. (2009) [24]. According to this, the resistance reduction increases almost in proportion to the riblet size when the riblet spacing is very small (the viscous regime), but depending on the shape, the viscous regime collapses after reaching its maximum near the s^+^ ≈ 10–20 and eventually transitions to the drag-increase regime, resulting in a structural collapse of performance when it is out of the optimal range. In addition, since comparing the optimum point to only s^+^ increases the dispersion by shape, this study proposes ${\left(A_{g}^{+}\right)}^{1/2}$ and shows that the optimum point can be better organized at the level of ${\left(A_{g}^{+}\right)}^{1/2}\approx 10.7\pm 1.0$. To substantiate this scaling and probe the breakdown mechanism, the authors conducted DNS of ribbed-channel flow, outlining the numerical approach and simulation parameters, and reported good agreement with the experimental drag-reduction envelope. Their DNS-based flow-field statistics further indicate that the viscous-regime breakdown coincides with the disappearance of the recirculating region inside the grooves; once it vanishes, Reynolds stresses over the grooves become increasingly important, leading to loss of riblet efficiency and eventually a net drag increase.

The CFD evidence in this paragraph spans a wide fidelity range, from review-level synthesis to LES/DNS and simplified RANS closures, and performance is primarily quantified through direct comparisons against smooth-wall baselines. High-fidelity approaches provide the most defensible mechanistic claims: Martin et al. (2016) [17] conducted transient LES in a periodic channel to directly compute time-averaged drag differences and to delineate a practical viscous-scaled window for robust riblet benefits, while Sasamori et al. (2017) [18] employed DNS with an immersed-boundary method and explicitly checked grid/domain sensitivity while benchmarking key smooth-wall statistics against prior DNS. Importantly, the regime-transition perspective summarized by García-Mayoral et al. (2009) [24] helps interpret why an optimal window exists and why performance can collapse outside it; by proposing a groove-area-based wall-unit scaling and supporting it with DNS that agrees with an experimental drag-reduction envelope, this work strengthens the generality of CFD-based design criteria beyond a single s^+^ optimum. In contrast, Wu et al. (2017) [19]) adopted a steady RNG k–ε model on a simplified 3D fish-scale geometry; although discretization/convergence settings and outlet boundary-condition sensitivity were checked and drag decomposition was reported, the turbulence closure does not resolve microscale flow physics within the surface relief. Therefore, the reported drag reduction is better interpreted as conditional feasibility rather than a transferable absolute value.

In summary, two constraints dominate translation to heat-exchanger channels. First, the reported optimal ranges are defined in wall units and thus shift with friction velocity; in practical heat exchangers where $u_{\tau }$ varies with Reynolds number, viscosity, and operating point, maintaining near-optimal s^+^/h^+^ would require either a narrow operating envelope or redesign across expected conditions. Second, riblet performance exhibits a regime transition with sharp off-design penalties: geometric changes that trigger separation or that eliminate stabilizing groove recirculation can rapidly increase pressure drag and Reynolds-stress-driven losses, reversing the net benefit. Together with the limited Reynolds-number ranges of many simulations, sensitivity to flow alignment reported in the broader riblet literature, and fabrication constraints such as low thermal conductivity in polymer-processed riblets, the current CFD body of evidence is most actionable for identifying trend directions and failure boundaries rather than certifying absolute savings. Accordingly, these drag-reduction textures should be prioritized when pumping-power savings dominate and the operating envelope is sufficiently stable to preserve the wall-unit optimum; otherwise, application should remain conservative unless Nu–ΔP co-validation and off-design risk mapping are performed under representative heat-exchanger conditions.

**Turbulence Promotion and Thermo-hydraulic Co-optimization**

Fish-skin-inspired surface designs demonstrate remarkable potential to overcome the inherent thermo-hydraulic trade-off, achieving simultaneous optimization of heat transfer performance and flow resistance. A representative example is the study by Gürel et al. (2023) [25], which applied shark-skin denticle structure to a plate heat exchanger using CFD to address its inherently high pressure drop problem. The denticle-inspired structure significantly enhanced flow mixing, resulting in a 50% increase in heat transfer rate, while its streamlined morphology minimized flow resistance, yielding an extraordinary 72.5% reduction in pressure drop. This outcome represents a rare and exemplary instance of simultaneous thermo-hydraulic optimization, where heat transfer enhancement was achieved without incurring a pressure-drop penalty, thus approaching the engineering ideal of performance balance in heat exchanger design.

To further maximize the effectiveness of bioinspired surface textures, a hybrid approach combining them with functional fluids has been actively explored. Liu et al. (2025) [26] applied a magnetic nanofluid in a shark-skin-structured tube to reinforce the properties of the surface texture and working fluid at the same time. This study numerically investigated a fish scale inspired bionic tube using a validated SST k–ω RANS framework with near-wall resolution control (y^+^ ≈ 1), and reported coupled changes in Nu and friction relative to a smooth-tube baseline. The overall thermo-hydraulic metric exhibited a non-monotonic Reynolds number dependence and peaked at 1.352 (Re = 16,840), which the authors attributed to vortex-induced boundary-layer thinning and enhanced near-wall mixing.

The precise optimization of geometric parameters is essential for the successful implementation of bioinspired surface structures. In the study by Yao et al. (2024) [27], a systematic CFD-based analysis of fish scale inspired internal textures in MCHEs identified a PEC > 1 design window by varying fin height and arrangement, and showed that excessive fin height can induce local stagnation and degrade overall performance, whereas a dense-front/sparse-rear distribution provides the most efficient configuration. Similarly, Liu et al. (2024) [28] performed turbulent-flow CFD of fish scale bionic enhanced tubes and reported a maximum PEC of approximately 1.332 under an optimized geometric combination, using a smooth-tube baseline to define the reference for normalized performance comparisons. These findings highlight the critical importance of geometric control in achieving performance optimization for bioinspired thermal systems.

Dey et al. (2019) [29] completed a customized design to meet the requirements of the system with only a single parameter adjustment of fish scale structures. In parallel, they built a three-dimensional conjugate heat-transfer CFD model ANSYS Fluent V16 and validated it against the experimental pressure drop, temperature, and Nusselt number before extending the analysis to parametric evaluation. By varying a single geometric parameter (the slope height), the design could be tuned either to achieve simultaneous improvement (14% higher heat transfer with 5% lower friction) or to maximize heat transfer by 2.05 times at the expense of a pressure-drop penalty, yielding a peak PEC of 1.76. The CFD results further explained that the heat-transfer gain stems from separation/recirculation and repeated thermal-boundary-layer disruption behind the scales, while the same wake structures can drive the pressure-drop penalty that binds the optimal operating/design window. This shows that the bioinspired texture provides high flexibility for customized design with only a single parameter adjustment.

Wang et al. (2025) [30] conducted a systematic comparison of channel textures inspired by crab shells, fish scales, and shark skin, analyzing their thermo-hydraulic performance under the same operating parameters. Using a CFD framework (ANSYS Fluent 20.0 with a k–ε turbulence model) and a representative single-channel setup based on geometric periodicity, they ensured a consistent basis for cross-texture comparison. The columnar protrusions of the crab-shell-inspired surface were found to not only generate vortices but also alter the direction of transverse velocity as the fluid bypassed the protrusions. The protrusion structure of these crab shells showed the best overall performance, improving the thermo-hydraulic composite performance (JF factor) by up to 15.02%. The superior performance was attributed to the ability of the crab-shell structure to effectively destroy the boundary layer while minimizing flow resistance, unlike the flow dead zones induced by fish-scale patterns. To support credibility, the authors reported mesh-independence and validated j and f factors against reference experimental data (average errors of 3.3% and 2.88%, respectively). This study clearly demonstrates that the careful selection of an appropriate biological prototype—aligned with the specific functional goals—plays a decisive role in determining the success of bioinspired texture design. Notably, the analysis focuses on the intercooler cold-side channel under idealized numerical conditions, so experimental and system-level confirmation remains necessary for direct design translation.

The performance of biomimetic designs can vary significantly depending on the chosen baseline for comparison. Hurry et al. (2024) [31] demonstrated that shark-denticle-inspired fins achieved an ideal simultaneous improvement—on average, a 19% reduction in pressure drop and a 13.1% increase in heat transfer when compared to conventional rectangular fins. These trends were obtained from a validated conjugate heat-transfer numerical framework, in which the baseline rectangular fin was experimentally characterized and used to calibrate the simulations for consistent cross-geometry comparison. However, when benchmarked against highly streamlined National Advisory Committee for Aeronautics (NACA) airfoil fins, the overall performance of the denticle fins was lower. In particular, despite its heat-transfer advantage, the denticle fin yielded a lower overall thermo-hydraulic factor (η < 1) relative to the NACA 0030 fin because the latter’s pressure-drop benefit dominates the composite metric. This finding underscores the critical importance of baseline selection in the evaluation and interpretation of biomimetic performance, as the perceived advantage of a nature-inspired design can depend strongly on the reference geometry used for comparison.

Zhao et al. (2025) [32] proposed an innovative hybrid microchannel design that integrates fish-fin-inspired wavy sidewalls with periodically arranged internal ribs. The fin-shaped sidewalls generated large-scale vortices in the main fluid flow, while the rib structures induced small-scale secondary flows near the wall, effectively disrupting the thermal boundary layer. As a result, the design achieved an exceptional maximum comprehensive performance index (η) of 1.82, indicating a significantly greater heat transfer gain relative to the pressure drop penalty. Notably, these conclusions were drawn from CFD-based numerical simulations supported by mesh-independence checks and a numerical-method validation against published experimental Nusselt-number data, which strengthens confidence in the reported trends. This study demonstrated numerically and experimentally that the intelligent integration of multiple biomimetic principles can yield higher performance potential than relying on a single biomimetic mechanism alone. For the turbulence-promotion and thermo-hydraulic co-optimization category, Table 3 summarizes the bioinspired prototypes by reporting heat-transfer gains and hydraulic penalties/benefits (each relative to the stated baseline), and by highlighting key validity limits and trade-offs across the references.

Across the fish skin-inspired turbulence promotion and thermo-hydraulic co-optimization studies, numerical consistency is generally maintained via mesh-independence and verification procedures, while the rigor of realism/validation varies substantially across references. For example, Liu et al. (2025) [26] employed a validated SST k–ω RANS framework with near-wall resolution control and reported benchmarked deviations using a smooth-tube reference, and Dey et al. (2019) [29] strengthened credibility by validating a 3D conjugate heat-transfer CFD model against experimental pressure drop, temperature, and Nusselt number prior to parametric evaluation. In addition, Wang et al. (2025) [30] reported mesh-independence and validated j and f factors against reference experimental data, providing a relatively robust basis for cross-texture comparisons under identical numerical settings. Nevertheless, the evidence level remains predominantly CFD-based, often relying on idealized channel representations, and validation incorporating fouling, durability, and manufacturability is still limited.

Moreover, because comprehensive indices (PEC, η, JF factor) and their defining equations/baselines differ across papers, absolute comparisons of reported maximum values are not straightforward; importantly, perceived superiority can be reversed depending on the chosen comparator. Therefore, for design translation, it is safer to interpret these findings as trend-level evidence under the stated baseline, and to prioritize explicit reporting of the baseline and metric definitions, the width of the operating window over which PEC/η remains over 1, and the collapse mechanisms that bound practical applicability. Accordingly, these textures are best justified in applications characterized by a relatively narrow operating envelope (Re/velocity), whereas separation- or stagnation-prone geometries warrant adoption only after robustness has been demonstrated across the anticipated operating range and representative non-idealities.

#### 3.2.2. Animal and Biological Systems

The inspiration drawn from biomimetic design extends beyond fish to include mammals, birds, insects, and other biological systems, each exhibiting unique thermo-fluid control mechanisms evolved to suit their respective habitats. These natural architectures often provide innovative solutions to specific engineering challenges that fish-inspired structures alone cannot fully address, demonstrating how the diversity of nature can greatly expand the horizons of engineering design possibilities.

Gürel et al. (2020) [33] proposed a plate surface pattern that simulates the bifurcation structure of human lungs, and suggested the possibility in CFD that it can simultaneously improve the heat-flow performance compared to the conventional Chevron pattern. Their CFD framework was benchmarked against a reference Chevron-type compact brazed PHE, which provides a basic level of credibility for the reported trends. The lung-inspired structure improved the heat transfer rate by 71.3% and reduced the pressure drop by 67.8% when compared with the conventional Chevron pattern. However, because the magnitude of the reported gains can depend on how strictly the reference geometry and operating conditions are matched, these values are best interpreted as indicative of the design potential rather than universally transferable. Since then, Gürbüz et al. (2023) [34] compared various biomimetic plate patterns such as fish gills, trachea, and lung-inspired architectures under the same conditions, showing that the lung-inspired pattern showed higher heat transfer performance (28% improvement compared to Chevron) than any other pattern and reaffirmed its potential as a key reference model in subsequent studies. At the same time, they noted that the more complex biomimetic channels can incur an increased pressure drop, highlighting an inherent thermo-hydraulic trade-off. However, because this ranking is established under a fixed CFD setup at a single operating point, it should be interpreted as a screening-level conclusion rather than a fully generalizable design rule. Although the authors report a Chevron-type reference comparison for numerical validation, the paper does not provide a quantitative error/uncertainty budget that would guarantee the same level of fidelity across all biomimetic geometries. However, since the conclusion of this step is basically based on numerical analysis, it is necessary to demonstrate whether the same trend is maintained in an environment including actual fabrication, surface roughness, and full-scale header/port-induced maldistribution effects in realistic PHE stacks, where small geometric deviations and distribution non-uniformity can dominate the net thermo-hydraulic outcome.

In this context, Göltaş et al. (2022) [35] integrally manufactured compact PHE applying the shape of open alveoli of the lungs to the plate surface via DMLS-based additive manufacturing and tested in parallel with CFD to verify how the shape proposed in the numerical analysis works in real hardware. To support the credibility of the numerical trends, they modeled the full flow passages including inlet/outlet ports and performed mesh-independence checks using an SST k–ω turbulence framework. The results show that the alveolar pattern can significantly increase the heat transfer/efficiency through flow disturbance, but at the same time, it was revealed that the increased turbulence in the groove and narrow flow paths can be accompanied by a significant pressure drop penalty. Notably, the experiment–CFD deviation in heat-transfer rate was reported to be on the order of 10% (trend-level agreement), while the pressure-drop penalty remained substantial relative to the classical chevron baseline. In other words, the ideal result of simultaneous enhancement of heat transfer and reduction pressure drop as shown in Gürel et al. (2020) [33] can vary in the actual implementation depending on the reference shape, manufacturing tolerance/roughness, and flow distribution (manifold) conditions between flow paths, and in Göltaş et al. (2022) [35] specifically suggests that system-level optimization including not only the pattern itself but also the port diameter, plate spacing, and guiding structure should be performed in parallel in practical design.

This awareness of the trade-off at the implementation stage naturally motivates the approach taken by Güler et al. (2024) [36] who advanced lung-inspired compact plate heat exchanger (CPHE) concepts to additive-manufacturing-based hardware tests while including the material (heat conductivity) itself as a performance variable. By fabricating lung-pattern CPHEs with identical geometry and roughness from steel, aluminum, and titanium powders and evaluating them against a conventional chevron-type brazed PHE, the study makes the respective contributions of geometry and material properties more separable than prior purely numerical comparisons. In parallel, a validated numerical model was used to interpret the flow/heat-transfer mechanisms observed in the experiments, strengthening the engineering relevance without relying solely on CFD. In other words, study in Güler et al. (2024) [36] can be said to have evolved in the direction of engineering the performance potential of the lung-inspired design by combining shape optimization and material selection based on the limitations of the implementation and manufacturing perspective shown by Göltaş et al. (2022) [35].

**Figure 4:**
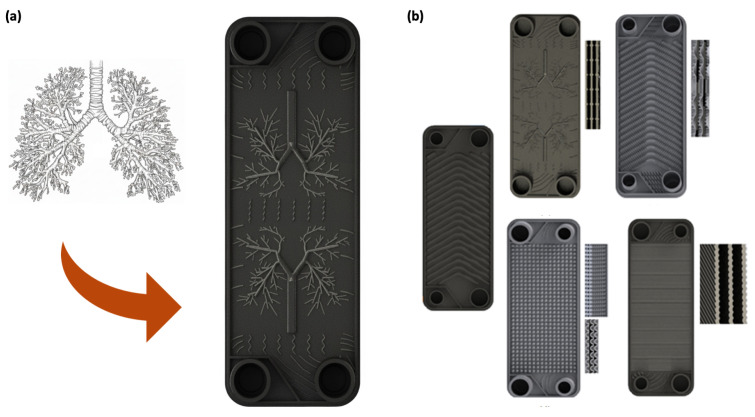
The lung pattern and its comparison with other plate heat exchanger surfaces (Texture Scale). (**a**) Schematic representation of the lung-inspired pattern (bronchial tree) and its location on the plate surface of a PHE. The design is inspired by the complex and efficient bifurcation architecture of the human lung. (adapted from [33]) (**b**) Comparison of various compact plate heat exchangers with different plate surface patterns, including those inspired by biological systems (e.g., lung-inspired) and conventional geometries (e.g., Chevron). The lung-inspired pattern aims to achieve a high heat transfer rate while minimizing pressure drop (adapted from [34]).

The success in these metallic materials has naturally led to the next study. Targeting applications that demand lightweight construction and corrosion resistance, Güler et al. (2025) [37] fabricated a lung-inspired plastic PHE using PA12 nylon and Multi Jet Fusion additive manufacturing technology. Experimental results confirmed the feasibility of this approach—despite being made of plastic, the PHE achieved a commendable maximum heat transfer rate of 3887 W, which is within a comparable order of magnitude to lung-patterned metallic prototypes reported at similar device scales and operating ranges. In addition, a CFD model validated against the measured outlet temperatures and pressure drops was used to interpret the internal temperature/pressure/velocity fields, supporting the conclusion that hydraulic losses grow disproportionately with increasing flow rate. However, the study also revealed a clear limitation: as the flow rate increased, the required pumping power rose nearly sixfold, leading to a sharp decline in the coefficient of performance (COP) (from 345 to 142). These findings demonstrate that while the lung-inspired structure retains its effectiveness even in lightweight polymer systems, the optimization of hydraulic losses associated with material property differences and additive-manufacturing-induced surface tolerance/roughness remains a crucial challenge for future development. This work thus delineates a clear research trajectory for advancing the practical application of polymer-based bioinspired heat exchangers. As illustrated in Figure 4, the lung-inspired branching topology was directly embossed onto the plate surface and realized as full-scale PHE prototypes, which were experimentally compared with conventional Chevron and other reference plate patterns.

Not all biomimetic designs lead to successful outcomes, and in many cases, design failures provide even more valuable engineering insights. Gadakh et al. (2022) [38] applied the delicate branching structure of bird feathers to a plate heat exchanger, assuming that such geometry would promote effective fluid mixing. However, CFD simulations showed a significant decline in heat transfer performance compared with the conventional Chevron pattern. To support the credibility of the comparison, the CFD setup was first validated against a reference Chevron PHE by matching outlet temperatures and heat transfer rate within a small deviation. The researchers attributed this to the occurrence of bypass flow, wherein the fluid avoided the densely patterned regions and instead traveled along the low-resistance edges, preventing effective utilization of the patterned area. Because this conclusion is derived from numerical evidence, it remains necessary to verify whether the same bypass-flow tendency persists when ports/manifolds and manufacturing tolerances are included. This is a representative counter example of how important it is to design system units that take into account the interaction with the entire flow path and pressure distribution beyond simply geometrically imitating the shape of nature.

The failure case of Gadakh et al. (2022) [38] shows that what makes it resemble a feather shape does not always guarantee the improvement of mixing/heat transfer, and Benschop et al. (2017) [39] support this intuition via DNS of turbulent channel flow over a bird-feather-inspired herringbone riblet texture. They quantitatively showed that the secondary flow formed by the convergence/herringbone riblet is not always advantageous, and when the span direction wavelength ($\frac{f}{L_{z}}$) is large, it is similar to the yaw behavior of the parallel riblet, showing only a slight decrease in drag of about 2% due to the suppression of turbulent momentum transport, whereas when ($\frac{f}{L_{z}}$) is reduced to O(1) and the influence of the convergence/scattering section increases, the drag increases by up to 73% because the strong secondary flow enhances both mean and turbulent advective transport. In other words, the assumption that adding the branching/convergence-scattering element will improve the mixing can result in the opposite flow penalty depending on the conditions, and even in PHE, if the dense pattern increases the local resistance/pressure field non-uniformity, the fluid can avoid to a path with small resistance, thereby strengthening the bypass flow. Therefore, the feather simulation pattern should be redefined as a thermal-hydraulic co-optimization problem that coordinates characteristic length (branch width/pitch) and convergence/diffusion strength together from a pressure drop-heat transfer perspective.

The inspiration drawn from biomimetic design has extended even into the insect world, and sometimes develops into a hybrid strategy that maximizes performance in combination with other physical phenomena. Tang et al. (2021) [40] applied a grooved bionic surface inspired by the wing microstructure of the oriental dragon louse to a liquid-cooled heat sink, effectively reducing air resistance. They further enhanced the system by introducing a magnetic nanofluid and an external magnetic field, which dramatically improved thermal dissipation performance. As a result, the Nusselt number—a measure of heat transfer efficiency—was enhanced by up to 163%, clearly demonstrating the powerful synergy that can be achieved through the integration of bioinspired surface textures with functional fluids in advanced thermal management applications. Notably, this work is primarily validated through experiments and post-processed performance indices rather than CFD-resolved flow-field evidence, so additional numerical analysis would be beneficial for generalizing the mechanism beyond the tested operating window.

**Figure 5:**
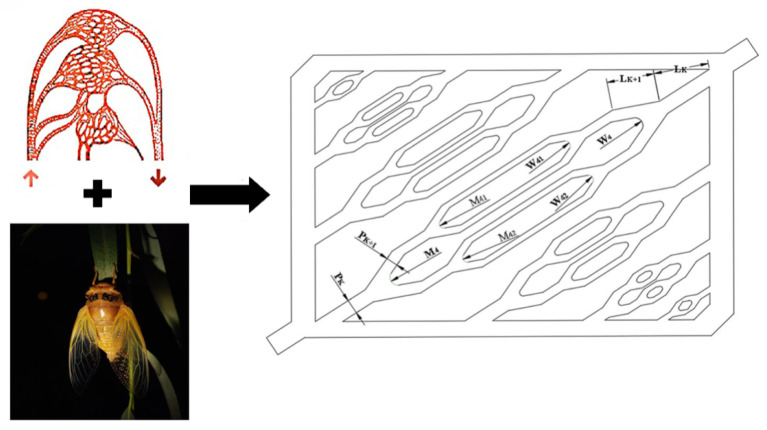
Examples of texture and hybrid network scale biomimicry. Schematic of a hybrid microchannel network design that integrates the structure of human blood vessels with the distribution pattern of insect wing veins. This composite architecture aims for uniform coolant distribution and minimized flow resistance in thermal management systems (adapted from [41]).

To meet the complex system-level requirements of modern thermal management, researchers have begun exploring hierarchical biomimetic designs that integrate multiple natural principles. Wang et al. (2024) [41] proposed an innovative cooling plate that combines the branching structure of human blood vessels with the distribution pattern of insect wing veins and evaluated it through coupled simulation and experiment to address two major challenges in electric vehicle battery systems—thermal runaway prevention and uniform cooling performance. The design features a hybrid bifurcation network, where gradually narrowing primary channels, resembling blood vessels, are coupled with widely distributed microchannels inspired by insect wing veins. This composite branching structure effectively ensures uniform coolant distribution across all battery cells in a design-intent sense, and the simulations show reduced temperature non-uniformity while quantifying the accompanying pressure-drop and pump-power trade-off. By sweeping coolant flow rate and the contact arc radius between channels, the study identifies an operating/design window where further cooling gains diminish while hydraulic penalties increase, thereby achieving the dual objectives of uniform cooling and flow resistance reduction. The study presents an effective system-scale biomimetic solution that achieves two conflicting goals simultaneously. As shown in Figure 5, the proposed cooling plate is designed by combining the branching pattern of blood vessels with the vein distribution of an insect wing to form a hierarchical channel network.

The wavy surface of squid fins, an adaptation found in invertebrates, offers a natural mechanism for enhancing fluid mixing. Yue et al. (2025) [42] applied this principle to microchannel design by integrating a snowflake-shaped flow network with a squid-fin-inspired wavy base plate, precisely controlling the amplitude and frequency of the surface waves. Their conclusions are drawn from 3D conjugate heat-transfer numerical simulations (steady laminar flow), supported by grid-independence and literature-based validation, which strengthens the credibility of the reported trends while remaining simulation-based. Their results confirmed that the design can maximize heat transfer under certain conditions and reduce pressure drop under others. Notably, the overall performance exhibited a clear design window rather than a monotonic trend, yielding a peak comprehensive index, demonstrating that the squid-fin-inspired surface can effectively contribute to thermo-hydraulic performance optimization by balancing heat transfer enhancement and flow resistance reduction. However, because the model treats the nanofluid as a simplified single-phase coolant and does not include fabrication-induced roughness or experimental uncertainties, the robustness of the optimal window should be re-examined under realistic manufacturing and testing conditions.

The CFD in the above studies generally presents heat transfer performance and flow resistance together and conducts CFD design and evaluation primarily based on relative comparisons using chevron or smooth channels as references. The effect of geometric biomimicry itself is reported as simultaneous improvement relative to chevron, and in Gürel et al. (2020) [33] for the lung pattern and in the DMLS-fabricated plate heat exchanger of Güler et al. (2024) [36], the directionality of shape optimization itself is revealed relatively clearly through CFD-based comparisons. However, if the performance metrics are extended to an energy perspective, in the plate heat exchanger optimization of Güler et al. (2025) [37], even if heat transfer performance is presented at a metallic level, it is difficult to conclude that the system is optimized solely by Nu (or h^+^) when the trade-off is considered, and the bird-feather pattern of Gadakh et al. (2022) [38] also shows a trade-off in which pressure drop decreases due to bypass flow while the heat transfer coefficient decreases, thereby revealing that consistency of the metrics is key to interpreting the results.

In terms of reflecting reality, the simplification of conditions and models remains a common limitation, and Gürbüz et al. (2023) [34] compared animal-inspired patterns but evaluated effectiveness and pressure drop under a single condition, and Yue et al. (2025) [42] analyzed a snowflake–squid-fin hybrid microchannel in COMSOL under assumptions that ignore multiple conditions, imposing constraints on absolute performance prediction. Nevertheless, since Wang et al. (2024) [41] also reported improvements by evaluating an insect-wing-vein/blood-vessel-network-based cooling plate through both experiments and numerical analyses, the CFD in this set is highly reliable for conditional relative comparisons, but when extending to real systems, it is reasonable to interpret the absolute performance values of the remaining designs on the premise of additional validation, using studies with experimental anchors as the reference axis.

In summary, heat exchangers inspired by animal and biological systems simultaneously design flow distribution and boundary-layer disturbance through branching/hierarchical flow paths, and, in some cases, combine functional fluids or magnetic fields to address heat transfer and flow resistance together. The performance-evaluation axes mostly place heat transfer performance (heat transfer rate, Nu, maximum temperature, etc.) and flow resistance (ΔP, friction factor, pumping power, etc.) side by side, and, when necessary, quantitatively compare the trade-off between the two axes using comprehensive indices such as PEC or COP. However, improvement does not come for free, and limitations associated with each pattern have been identified, such as increased flow resistance depending on conditions, including increases in flow rate and the angle of the magnetic field. In particular, there exists a failure case in which the bird-feather pattern can instead deteriorate heat transfer due to bypass flow if it fails to control the overall pressure distribution of the flow path. Therefore, in practical design, it is reasonable to prioritize application when operating Re and flow-rate ranges can be maintained within the optimal pattern, but under conditions with large flow-rate fluctuations or steep ΔP and viscosity penalties at high flow rates, validation using comprehensive indices is required.

#### 3.2.3. Plants and Natural Phenomena

The sources of biomimetic inspiration extend beyond the animal kingdom to include plants, waves, and even inanimate natural phenomena such as snowflakes. These alternative approaches often reveal previously unexplored physical mechanisms that are absent in conventional animal-inspired studies, offering novel pathways for improving not only convective heat transfer but also complex phase-change processes such as condensation and evaporation.

The flow patterns of natural phenomena such as ocean waves, though non-biological, can also inspire innovative surface designs for heat exchangers. Gürbüz et al. (2023) [34] developed a surface pattern that mimics the complex and periodic motion of waves, incorporating geometrical undulations that periodically alter the flow path of the working fluid as it moves through the channel. This wavelike geometry is expected to induce secondary flows and enhance cross-stream mixing, which the authors summarize as increased turbulence, perpendicular to the main flow direction. These flow structures effectively disrupt the stagnant thermal boundary layer near the wall—the primary source of thermal resistance—while simultaneously enhancing mixing between the hot near-wall fluid and the cooler core flow. Numerical comparisons with a conventional Chevron pattern revealed that the wave-inspired design achieved a 12% higher heat transfer rate under the stated CFD conditions, along with the general observation that biomimetic/narrow channels tend to raise pressure drop. This shows that the dynamic motion of natural phenomena can effectively inspire static structure design with enhanced thermal efficiency. At the same time, because the mechanism is not substantiated here with detailed field-level evidence and the conclusion is drawn at a fixed operating condition, the claimed benefit should be treated as conditional until validated under realistic manufacturing roughness and stack-level flow distribution.

The fractal branching geometrical structure found in natural phenomena such as snowflakes offers an ideal spatial solution for achieving uniform fluid distribution with minimal loss within confined domains. Yue et al. (2025) [42] adopted the symmetrical sixfold branching pattern of snowflakes as the flow network of a microchannel cooling plate, optimizing both the inlet and branching angles to reduce nonuniformities in flow and temperature distribution. In particular, their angle analysis links temperature nonuniformity to local dead zones and motivates a geometry arrangement that enforces a consistent inflow structure alignment across branches. As a result, the optimized configuration of the composite design reports an overall heat-dissipation improvement up to 32.56% with a maximum PEC of 1.68, illustrating how a snowflake-like branching network can serve as an efficient backbone for system-wide thermo-hydraulic optimization when integrated with complementary surface-level enhancements. Nevertheless, because the study focuses on idealized channel domains rather than full manifolds/packaging-induced maldistribution, the claimed uniformity benefits may shift once headers and assembly-level flow distribution are included.

In these studies, CFD is primarily used as a screening and parametric optimization tool for ranking complex biomimetic flow paths under identical boundary conditions, but the rigor of validation varies substantially across references. In the lung-inspired PHE literature, purely numerical claims of simultaneous heat-transfer enhancement and pressure-drop reduction should be interpreted cautiously until they are supported by hardware-level evidence that includes stack-relevant flow passages. In this regard, the experiment-coupled CFD studies that model full passages including inlet/outlet ports, report mesh-independence, and benchmark predicted trends against measured heat-transfer and hydraulic data provide a more defensible reference axis for interpreting subsequent CFD-only designs. Conversely, studies that rely on a single operating point or present limited quantitative validation/uncertainty information remain most reliable for conditional, relative comparisons rather than absolute performance predictions.

From a critical-design perspective, the studies in these subsections consistently indicate that the reported improvements are strongly governed by an operating/design window rather than a monotonic trend. Wave-inspired undulating channels and snowflake-like branching networks can enhance mixing or distribution and thus reduce thermal resistance, yet the same geometric complexity tends to introduce narrow passages and additional turning losses that readily translate into pressure-drop penalties once the flow rate increases or the alignment/arrangement deviates from the assumed configuration. Likewise, hybrid architectures (e.g., branching networks combined with wavy surfaces) may exhibit an optimum window in which thermo-hydraulic indices peak, while off-design conditions can rapidly erode the net benefit. Therefore, for practical deployment, these CFD-derived conclusions should be carried forward with multi-point validation across realistic operating ranges, inclusion of header/port/packaging-driven maldistribution effects, and explicit consideration of fabrication-induced roughness and uncertainty so that the design window remains robust under real manufacturing and system constraints.

In texture optimization, non-ideality of manufacturing and experiments is not just a practical consideration but a key variable that changes performance conclusions. For example, in the experiment Benschop et al. (2018) [43] of riblet coating, Taylor–Couette torque measurements showed that data scatter can arise from low-shear torque uncertainty and small differences in coating thickness, surface smoothness, cylinder alignment, and gap/geometric deviations, suggesting that the tolerance and measurement uncertainty budget should be designed together when the CFD/DNS-based optimal shape is actually produced. Furthermore, since fine textures can weaken or lose the hydrodynamic benefits by increasing biofilm accumulation under low shear or stationary conditions, Benschop et al. (2018) [43] explicitly reports that riblets may show no clear benefit under stationary conditions because biofilm settlement/growth can increase, the texture design should be verified from the perspective of failure modes and windows with contamination/degradation involved, rather than a single operating point of a clean surface assumption.

#### 3.2.4. Texture Scale Critical Synthesis and Design Guidelines

The core of texture-scale biomimicry lies in resolving the thermo-hydraulic trade-off under realistic constraints rather than in reporting an isolated peak-performance point. Across the studies reviewed in Section 3.2, CFD is the primary engine for parametric exploration and mechanism elucidation; however, the credibility and transferability of CFD outcomes are fundamentally conditional on the validation strength, baseline consistency, and operating-point representativeness.

First, even when texture designs are expressed in wall units to improve generality, the governing wall-unit variables depend on the friction velocity $u_{\tau }$ and therefore shift with Reynolds number and channel-specific shear conditions. Consequently, a geometry optimized at a single operating point can become off-design as the operating condition changes, unless robustness is explicitly verified over the expected shear/flow range rather than assumed from one optimal condition.

Second, many studies maintain internal numerical consistency, and several provide experimental anchors that support relative comparisons under the stated baseline. Nevertheless, because comprehensive indices (PEC, η, JF factor) and their comparison baselines are not standardized across papers, cross-study comparisons based on a single maximum PEC value are intrinsically fragile; depending on metric definition and reference selection, the same outcome can be over- or underestimated in interpretation. Therefore, it is safer to interpret results as trend-level conclusions under identical baselines, particularly for setups with weak validation frameworks, correlation-based validation, or strong boundary-condition sensitivity.

Third, for complex passages such as plate heat exchangers and hierarchical channels, an additional gap emerges between idealized CFD domains and deployable systems. Realistic performance can be dominated by fabrication-induced roughness/tolerances and by full-scale header/port-induced maldistribution in practical stacks; thus, single-condition CFD rankings of surface patterns should be treated as screening-level evidence unless they are re-evaluated in system-representative domains (including ports/headers) with an explicit uncertainty/tolerance budget. In this context, system-level co-optimization—including not only the pattern itself but also port diameter, plate spacing, and guiding structures—becomes necessary for practical design translation.

Accordingly, non-ideality of manufacturing and experiments is not merely a practical nuisance but a performance variable that can change performance conclusions. For example, in the experiment by Benschop et al. (2018) [43] on riblet coatings, coating thickness, surface smoothness, cylindrical alignment and geometric deviation as well as torque measurement uncertainty are pointed out as the cause of the data distribution, suggesting that the tolerance and measurement uncertainty budget should be designed together when the CFD-based optimal shape is actually implemented.

Ultimately, the critical synthesis of the texture scale indicates that the key question is not “which texture is always superior” but “under which objectives and constraints performance is maintained despite operating fluctuations.” Therefore, texture-based performance claims should report separated heat-transfer gain and hydraulic penalty/benefit under the same stated baseline, the operating/design window over which PEC > 1 is maintained, and the points and causes at which performance collapses to PEC < 1. Validation should be designed not only to reproduce maximum values but also to identify when and how failure modes occur under non-ideal factors such as tolerances, fouling, and wear, enabling reproducible and transferable comparisons.

Table 4 summarizes the texture scale bioinspired technologies discussed above as a design decision-making framework. The Works/Fails entries consolidate the conditions in which each approach is effective and the main real-world risk drivers, which vary with flow regime, geometry/manifold design, manufacturing constraints, and fouling/icing rather than indicating an absolute ranking. In addition, based on each referenced work, the key evaluation metrics are presented as recommended performance indicators to improve cross-study comparability and real-operating-condition reproducibility.

### 3.3. Network Scale

The studies discussed thus far have introduced various bioinspired techniques that enhance the efficiency of heat exchangers without altering their overall operating principles. This section focuses on technologies that mimic the complex and efficient heat and mass transfer systems found in nature to further improve heat exchanger performance. These approaches fundamentally modify the structure and operating mechanisms of heat exchangers, resulting in a diverse range of configurations and, consequently, different methods of performance evaluation.

#### 3.3.1. Triply Periodic Minimal Surface (TPMS)

**Figure 6:**
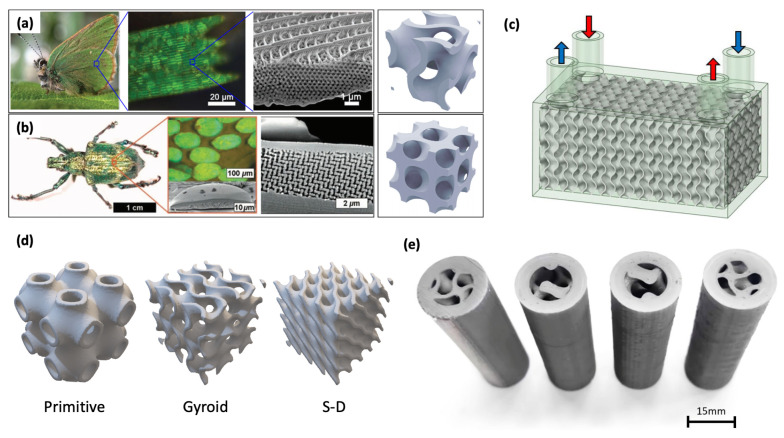
TPMS structures inspired by nature (Network Scale). (**a**) Images showing the photonic crystals of a butterfly wing scale and a computer-generated model of the Gyroid TPMS structure. (**b**) Images showing the scales of the weevil Lamprocyphus augustus and a computer-generated model of the Schwarz-D TPMS structure. (**c**) Example Application of a TPMS Structure in a Heat Exchanger. Red and blue arrows denote the hot- and cold-fluid streams, and the arrow directions indicate the flow directions. (**d**) Design models of TPMS test arrays, including the Primitive, Gyroid, and Schwarz-D geometries. (**e**) Example of 3D-printed cylindrical tubes filled with a sheet-type Gyroid lattice, demonstrating TPMS application in heat exchanger cores ((**a**,**b**) adapted from [44], (**c**) adapted from [51], (**e**) adapted from [46]).

The triply periodic minimal surface (TPMS) represents a mathematically formulated bicontinuous structure observed in natural systems such as the photonic crystals of butterfly wing scales, the cubic phases of cellular biomembranes, and the scales of beetles, as shown in Figure 6a,b. Mathematically, a TPMS is defined as an infinitely periodic minimal surface with zero mean curvature, and its representative structures—G (Gyroid), D (Schwarz–D), and P (Schwarz–P)—were systematically categorized in Schoen’s classical report. The continuous and smooth surfaces of TPMS architectures offer engineering advantages in heat exchanger design, including enhanced surface-area-to-volume ratio and the promotion of mixing and vortex generation, as illustrated in Figure 6c. This section introduces research that mimics naturally occurring TPMS structures to improve the thermal performance of heat exchangers.

In the supercritical CO_2_ Brayton cycle context, Li et al. (2020) [44] introduced biomimetic channel designs incorporating TPMS structures inspired by the Gyroid form observed in butterfly wing scales and the Schwarz-D geometry found in the weevil Lamprocyphus augustus. Notably, the strong flow mixing generated within the TPMS channels produced substantially higher turbulent kinetic energy than that in conventional PCHEs, leading to an increase in the Nusselt number (Nu) by 16% to 120% under equivalent pumping power. This benefit comes with a hydraulic penalty: the Gyroid and Schwarz-D channels exhibit friction factors about 50–100% higher than the PCHE baseline. Using the PEC referenced to the PCHE, both TPMS designs achieved a net thermo-hydraulic improvement of 17–100%. Nevertheless, since this study was limited to CFD simulations with sCO_2_ as the working fluid, further investigations are required to ensure structural reliability during actual manufacturing and to conduct experimental validation of the proposed TPMS-based heat exchanger design.

Liang et al. (2023) [45] experimentally implemented various TPMS topologies in practical heat exchanger designs. Using additive manufacturing based on selective laser melting, they fabricated three aluminum alloy heat exchangers with Schwarz-D (S-D), Gyroid, and Primitive geometries, as shown in Figure 6d, and evaluated their flow characteristics and heat transfer performance through both experiments and numerical simulations. Simulation results revealed that the S-D and Gyroid structures generated three-dimensional flow patterns and large-scale bifurcating streams owing to their helically curved flow channels. In particular, the S-D configuration exhibited a distinct counter-flow effect, leading to a significant enhancement in heat transfer. Among the tested designs, the S-D heat exchanger demonstrated the largest heat transfer surface area and the most complex flow pattern, achieving 41–52% higher heat transfer rates than the Primitive heat exchanger and 4–12% higher rates than the Gyroid heat exchanger. Since the surface-to-volume ratio of TPMS structures increases rapidly with decreasing unit cell size, future optimization studies—such as reducing the current unit size of 16 mm—are expected to further maximize the thermal performance potential of TPMS-based heat exchangers.

Dassi et al. (2024) [46] proposed an alternative approach that differs from conventional TPMS topologies by filling cylindrical tubes with biomimetic Gyroid (TPMS) lattices. As shown in Figure 6e, two configurations were developed—sheet-type and solid-type Gyroid structures—and their heat transfer rates, pressure drops, and turbulence intensities were compared through both numerical simulations and experimental measurements under varying fluid types and relative densities. Simulation results indicated that the Overall Enhancement Factor, defined as (Q/Q_0_)/(ΔP/ΔP_0_), was superior for the solid-type structure and showed an approximately inverse linear relationship with relative density, with the solid 15% model exhibiting the highest overall efficiency. Future optimization studies should incorporate additional factors such as coolant properties, operating conditions, and thermal conductivity variations associated with wall materials to further enhance the performance of Gyroid-filled TPMS heat exchangers.

Chen et al. (2023) [47] did not treat the Gyroid structure as a simple uniform geometry, but proposed a multidimensional gradient design that combines a level-set value gradient and a thickness gradient. According to the numerical results, the Gyroid with a level-set gradient exhibited an improved combination in terms of the heat-transfer–pressure-drop trade-off, with the convective heat transfer coefficient increased by up to 60.10% and the pressure drop reduced by up to 18.05% compared with the uniform Gyroid. This is attributed to thermal boundary-layer compression induced by the spiral motion generated by the Gyroid. They also identified an operating window of 0.2–0.3 mm in thickness and Re = 100–125 that can simultaneously satisfy high heat transfer and low pressure loss. However, since this study is mainly based on CFD numerical validation, additional experimental validation and multivariable optimization are required for practical implementation, including geometric reproducibility in additive manufacturing, material thermal conductivity/surface condition, and variations in operating conditions.

Lai et al. (2025) [48] realized a compact, high-performance heat exchanger required in the aerospace field by using a Gyroid-based TPMS lattice and fabricating it via additive manufacturing. They also evaluated the thermal response by conducting CFD studies under various inlet flow-rate and temperature conditions. The experimental results showed that the Gyroid heat exchanger achieved a 73.28% increase in heat transfer rate compared with a conventional plate heat exchanger under the same conditions. Across the tested flow rates, the CFD predictions of both $Q_{\mathrm{avg}}$ and the overall heat transfer coefficient U agreed with experiments within ±15%, thereby demonstrating the reliability of the model. Pressure drop increased with flow rate, with only a small hot–cold-side difference (≈0.82–1.76%). Meanwhile, in a material-effect comparison, the aluminum heat exchanger exhibited an additional 6.37% improvement in heat transfer rate relative to SS316L, which is interpreted as a result of the structural surface-area advantage combined with the higher thermal conductivity of aluminum. In the future, expanded-condition studies and optimization are needed, including wall-thickness reduction and the effects of fine surface roughness or fabrication deviations on heat transfer.

TPMS is a mathematical model that allows for straightforward parametric representation, making it not only a biologically inspired design but also a target for mathematical optimization. For instance, Yan et al. (2024) [49] fabricated a Gyroid-structured metallic heat exchanger via metal 3D printing and established experimental and numerical correlations, confirming that the measured heat transfer coefficient was approximately 9.7% higher than the theoretical prediction. Hu et al. (2025) [50] conducted optimization studies across various TPMS geometries, including Gyroid, Primitive, and Diamond structures, and found that the Diamond-type was most efficient under high-velocity flow, while the Gyroid-type performed best under low-velocity flow conditions. Additionally, Oh et al. (2025) [51] proposed a design method to simultaneously enhance flow uniformity and heat transfer performance by applying three types of functional gradation—filtering, cell-size variation, and level-set modulation—to TPMS-based heat exchangers. Collectively, these studies demonstrate that TPMS architectures, through mathematical modeling and experimental validation, have evolved into a distinct and promising class of heat exchanger design.

Sometimes, multiple types of TPMS structures are combined for use. Qian et al. (2025) [52] selected, among basic TPMS types, the low-resistance Gyroid and the high-heat-transfer Diamond, and then designed a Gyroid–Diamond hybrid TPMS core to combine the advantages of the two structures based on field synergy. They also fabricated specimens by 3D printing and compared heat transfer rate and pressure drop using CFD and two-fluid experiments. The experimental results showed a maximum heat transfer rate of 4827 W under a flow rate of 15 L/min and a hot-side inlet temperature of 50 °C, demonstrating superiority relative to the Gyroid and Diamond baselines. Across the tested flow-rate range, the CFD results indicated that the heat-transfer performance ranked as G–D > Diamond > Gyroid, with the G–D hybrid achieving 19.6–31.3% higher heat transfer than Gyroid and 2–11% higher than Diamond. The pressure-drop trend was Gyroid < G–D < Diamond, and the hybrid still reduced pressure drop by 5.0–17.9% versus Diamond, although transition-region acceleration, rotation, and stagnation introduce local losses. Future work should optimize the transition geometry to reduce local losses and extend the concept to multi-TPMS hybrids.

In many TPMS numerical studies, numerical convergence is treated in a relatively rigorous manner. However, manufacturing and experimental realism (surface roughness, printing defects, wall-thickness/geometry deviations) is not consistently represented, which can undermine validation of absolute performance values. Yan et al. (2024) [49] explicitly stated that surface roughness induces discrepancies between experimental and theoretical performance, making it difficult to directly validate simulations using only in-house experiments, and Dassi et al. (2024) [46] pointed out that, for specimens with a relative density of 15%, printing defects can increase the error. Conversely, some recent experimental studies report reasonable simulation–experiment agreement for selected geometries and operating windows, and in some cases argue that surface roughness or dimensional deviations are sufficiently small to be neglected. Ultimately, the TPMS CFD cited in this section is most trustworthy for discussing, under identical models and boundary conditions, the directionality of how topology, cell size, or grading affects the pressure-drop–heat-transfer trade-off, and for ranking the relative superiority among designs. However, generalizing absolute performance (U, Nu, Q, and ΔP) and interpreting scale-up remain limited, because surface roughness, manufacturing defects, and direct experimental validation of heat-transfer metrics is frequently incomplete.

The core performance-enhancement mechanisms of TPMS-based heat exchangers are increased heat transfer area and enhanced mixing promoted by three-dimensional interconnected pathways. Through these mechanisms, under identical operating constraints (i.e., the same flow rate or the same pumping power), one or more heat-transfer metrics among Nu, U, and Q were shown to be enhanced. However, this improvement is structurally accompanied by a friction-loss penalty, and some studies report practical fabrication limitations, such as manufacturing deviations, surface roughness, and increased wall thickness. In addition, many studies are CFD-only, and experiments have also been conducted mainly under limited conditions, resulting in insufficient validation for real environments and long-term durability. Therefore, from a practical design perspective, TPMS can be effective in conditions where pumping power is fixed and heat-transfer performance is prioritized, and in certain operating regimes where Q/ΔP is improved, but it should be applied conservatively in systems with manufacturing scenarios that cannot manage roughness or wall-thickness increases, and in topologies such as sheet-type designs where pressure drop can be large.

#### 3.3.2. Leaf Veins & Branches

The vein and branch networks found in leaves and trees are well known as natural transport structures that efficiently diffuse water and nutrients from concentrated regions to surrounding areas. Inspired by this mechanism, engineering designs that mimic such diffusion pathways have been widely adopted in heat sinks and other thermal management systems due to their exceptional heat dissipation performance. This concept can also be effectively extended to the flow channel network design of heat exchangers. This section introduces research that mimics the branching structures of leaf veins and tree branches to enhance the thermal performance of heat exchangers.

**Figure 7:**
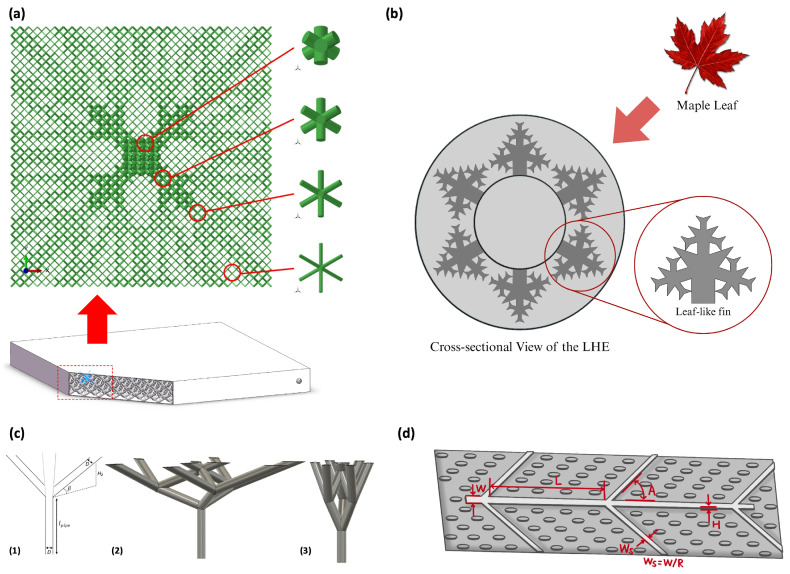
Flow network designs inspired by leaves, branches, and fractal geometry (Network Scale). (**a**) Overall morphology of a biomimetic lattice structure for fluid distribution, inspired by the leaf-vein pattern and realized by mapping onto a body-centered cubic lattice cell with a spatially graded lattice density. (**b**) Schematic diagram of a bioinspired latent heat energy storage system, featuring leaf-like fins inspired by the branching structure of a Maple leaf, designed to enhance the heat transfer performance of PCM. (**c**) Computational models of a three-level trifurcating 3D pipe network, mimicking the branching architecture of tree limbs: (1) definition of the branching angle, (2) representative branched geometries, and (3) corresponding network models used for heat-transfer analysis. (**d**) Schematic diagram of a leaf-vein-inspired rib network embedded within a microchannel for thermo-hydraulic optimization ((**a**) adapted from [53], (**c**) adapted from [55], (**d**) adapted from [56]).

Deng et al. (2021) [53] proposed a design that mimics the leaf-vein pattern for fluid distribution by introducing a spatially graded lattice density within a heat exchanger. Figure 7a shows the overall morphology of the biomimetic structure. The leaf-vein model was mathematically defined and mapped onto a body-centered cubic lattice cell, where the radius of structural cylinders was adjusted to gradually vary both the flow passages for the working fluid and the local density of the heat exchanger. As a result, a graded-density lattice structure with superior thermal diffusion characteristics compared to a uniform lattice was realized. Under two simulated working conditions, this biomimetic lattice structure transferred the same amount of heat that the uniform lattice released over 1200 s in only 85 s, reducing the required time to approximately 7% of the baseline case. However, since these findings are based solely on numerical simulations, further studies are needed to verify the manufacturability and structural reliability of the proposed design.

PCM-based thermal energy storage systems offer a promising solution to overcome the intermittency of clean energy by enabling high-density heat storage. However, their low heat release rate often limits system efficiency, as the phase transition process tends to occur slowly. To address this issue, Jiang et al. (2023) [54] developed a leaf-vein-inspired heat exchanger designed to enhance the heat release performance of PCM systems, as shown in Figure 7b. Their study demonstrated that the leaf-vein-mimicking fins reduced the solidification time of PCM by 41.6% and improved the heat dissipation performance by 69.9%. Based on a two-dimensional numerical model, they showed that solidification proceeds through a brief early convection-dominated stage followed by a predominantly conduction-dominated regime, yielding largely axisymmetric phase-interface and temperature fields for most of the discharge process. Nevertheless, to enable industrial application, further investigations on long-term stability, system reliability, and scalability are necessary to support the commercialization of this biomimetic design.

Mardanov et al. (2025) [55] designed a three-level trifurcating 3D pipe network that mimics the branching architecture of tree limbs, proposing it as a potential replacement for conventional vertical pipes in heat exchangers. Using numerical simulations, they investigated how variations in the branching angle (20°–65°) influence overall heat transfer performance. Figure 7c presents the Nusselt number results obtained to examine the variation in heat transfer performance as a function of the branching angle. The analysis revealed that introducing a branching network significantly enhanced heat exchanger performance, with the Re-normalized thermal performance factor (TPF) reaching its maximum at a branching angle of 36°. CFD analysis linked the optimum at 36° to the lowest mean normalized turbulent viscosity (µt/Re) yet the highest mean normalized vorticity (ΩU/D), suggesting heat-transfer enhancement dominated by organized rotational structures rather than dissipative turbulence. The study suggests practical potential, particularly for sealed low-fouling loops or aerospace systems where cleaning constraints differ. However, since the relationship between TPF and branching angle was shown to be non-monotonic, the results suggest that achieving optimal thermal performance for real-world applications will require precise geometric optimization of the branching network, including the branching angle configuration.

Conventional airfoil and zigzag channel heat exchangers exhibit a clear trade-off between enhanced heat transfer and increased pressure drop. To overcome this limitation, Zhang et al. (2025) [56] introduced a leaf-vein-inspired rib network—comprising main and secondary veins—embedded within an airfoil-shaped microchannel, and systematically compared its thermal–hydraulic characteristics through both experiments and CFD simulations, as shown in Figure 7. The results showed that secondary vein ribs significantly enhanced heat transfer by inducing boundary-layer disruption and vortex generation, whereas main vein ribs caused flow nonuniformity and higher flow resistance, thereby reducing overall performance. After parametric optimization, the best case achieved Nu = 65.27 and PEC = 1.26, corresponding to an 85% increase in Nu and a 26% increase in PEC relative to the baseline airfoil channel. However, geometric parameters such as rib height and diameter ratio were found to be highly sensitive to pressure drop, imposing design constraints. Moreover, since the results were obtained within a specific experimental range, further validation involving scale-up and refrigerant substitution is necessary to confirm the general applicability of the proposed design.

Wang et al. (2019) [57] used 3D printing to fabricate Y-type and H-type fractal-tree heat exchangers, as well as a conventional spiral-tube heat exchanger, under the same heat-transfer-area condition. Based on this, they compared the thermo-hydraulic performance through experiments and numerical simulations. As a result, the pressure drop increased with increasing flow rate, and overall the pressure drop of the spiral-tube was significantly larger than that of the two fractal-tree structures. CFD further showed that the Y-type maintained robust flow distribution (maximum terminal-channel deviation 3.76–12.31%), whereas the H-type suffered severe maldistribution (35.92–47.51%), consistent with the observed flow blind spots. In terms of heat transfer, the total heat flux was the highest for the H-type, while the Y-type and the spiral-tube showed similar values. However, the authors point out, through visualization experiments, that minor defects such as increased inner-wall roughness and installation errors can induce flow non-uniformity and flow blind spots, leading to deviations between experiments and simulations and to performance degradation. Further work is needed to secure structural robustness from a manufacturing perspective.

Calamas et al. (2015) [58] proposed a single-fluid compact solid heat exchanger using tree-like flow passages to mitigate the trade-off between heat transfer and pressure drop. The experimental apparatus was fabricated as a stacked structure inside a cylindrical housing, consisting of four aluminum tree-like disks with a hierarchical pattern and three spacer disks separating them. According to the analysis results, the pressure drop increased strongly with increasing flow rate, and a correlation of $\Delta P=1.185\;\cdot \;Q^{1.876}$ was presented for the overall heat exchanger. In terms of heat transfer, Nu increased significantly as Re increased, and Nu was organized linearly with respect to Re and correlated as Nu = 0.839·Re + 18.468. However, this study has analytical constraints, such as not considering the wall temperature, and since it is limited to unmeasured heat losses, a single material, and a single-fluid configuration, further optimization and validation are needed in the future, including material thermal conductivity/wall conditions, quantification of heat losses, multi-fluid operation, and geometric scaling variations.

Rastogi et al. (2024) [59] proposed a systematic design-improvement procedure for a tree-branching-inspired microchannel heat sink that aims at more uniform cooling without excessively increasing geometric complexity. Specifically, they tuned the design by inserting cylindrical pin fins at the branching junctions to control flow distribution and by converging the channels with a small angle. Using numerical analysis interpreted via the Overall Performance Index and entropy generation, the results showed that pin-fin insertion alone reduced the base temperature by 2.45%. At a dimensionless pin diameter of $d/D_{h}=1.25$, the branch flow rate ratio approached unity, and this condition was presented as the optimum in which the flow is most uniformly distributed. Channel convergence further reduced the base temperature by 2.71%. CFD also showed that the pin fin induces a recirculation zone at the junction, which increases pressure drop despite improving cooling uniformity.

Several leaf vein/branch-type numerical studies demonstrate computational reliability in implementing local mechanisms, particularly when near-wall resolution, mesh-independence tests, and validation references are explicitly reported. Zhang et al. (2025) [56] secured computational reliability by explicitly presenting y^+^ < 1 near-wall resolution and mesh-independence/validation procedures in a steady-state analysis. Mardanov et al. (2025) [55] specified a concrete CFD setup that directly compares a 3D pipe network with a vertical pipe, and treated junction flow as the key mechanism. In particular, they presented a comparison with empirical correlations for the vertical pipe as a validation reference and explained the sharp local increase in Nu, thereby securing reliability. Taken together, the numerical results in this section are highly reliable for relative comparisons under identical boundary conditions/metrics (optimization of angle, ribs, and number of branches) and for presenting local mechanisms such as boundary-layer disturbance. Likewise, some CFD-only design studies explicitly specify convergence criteria while relying on simplifying assumptions (single-phase, incompressible laminar flow; neglecting gravity/contact resistance/radiation), which should be acknowledged when interpreting absolute performance. However, many simulations still adopt idealizations (steady-state operation, prescribed wall-temperature/heat-flux boundaries, and omission of manufacturing-induced roughness or geometric deviations), so additional validation under realistic boundary conditions and as-built geometries is required for absolute/scale-up interpretation.

Accordingly, leaf- and branch-inspired networks are realized through diverse engineering-dominant mechanisms, such as spatially non-uniform flow-path distribution, vortex formation and flow redistribution, and increased heat-transfer area, depending on the objective and the mode of implementation. Development has mainly proceeded toward improving either convective heat-exchange frameworks (Nu, PEC, pressure drop) or phase-change and transient frameworks (solidification time, heat dissipation rate), with some studies additionally adopting combined indices such as OPI that explicitly couples Nu and friction penalties. However, these improvements are generally accompanied by penalties; in improving convective heat exchange, ribs or branching compress and non-uniformize the flow paths, increasing the friction factor. In addition, for phase-change and transient frameworks, the reported performance is largely based on numerical analyses, leaving structural reliability and manufacturability as gaps. Therefore, such bioinspired designs are most compelling where local mixing and boundary-layer disruption dominate, but for applications under tight ΔP budgets or limited manufacturing control, design selection should be based on constraint-aware multi-metric optimization (PEC/TPF/OPI as appropriate) and must be supported by scale-up, durability, and tolerance-aware validation rather than extrapolating absolute CFD values.

#### 3.3.3. Lung Network

The lung features a branched bronchial network interconnected with alveoli, providing a large effective surface area and uniform flow distribution even under short residence times. This biological principle can be translated into heat exchanger design by constructing bicontinuous or interwoven flow networks, which maximize the heat transfer area per unit volume while minimizing pressure drop. This section introduces studies that apply lung-inspired branched network architectures to the flow channel design of heat exchangers.

A desiccant-coated heat exchanger is a device in which the heat exchange surface is coated with a solid desiccant material, enabling the efficient dissipation of adsorption heat through internal cooling channels during the dehumidification process. Conventional designs—based on fin-and-tube, foam, or packed-layer configurations—exhibit a clear trade-off between adsorption area and pressure drop. To overcome this limitation, Puttur et al. (2022) [60] developed a lung-inspired heat exchanger that replicates the branched bronchial network, forming an intertwined bicontinuous flow network for air and cooling water using 3D printing. The thermal and mass transfer characteristics of the adsorbent-coated lung-inspired heat exchangers were experimentally evaluated, revealing an optimal adsorption rate of 54.8 g·m^−3^·s^−1^ and a pressure drop penalty of approximately 4 kPa, demonstrating a well-balanced enhancement between performance and flow resistance. However, the reported performance was limited by the experimental conditions and the SLA polymer material used in fabrication. Future studies focusing on system-level integration, material innovation (e.g., MOFs), and design optimization are expected to further advance this concept into a more efficient and scalable architecture.

Ahmadi et al. (2023) [61] proposed a ceramic heat exchanger design inspired by the alveolar architecture of the human lung to achieve high thermal efficiency under extreme temperatures exceeding 1000 °C, as required in next-generation supercritical carbon dioxide and concentrated solar power plants. The proposed topology forms two intertwined bicontinuous air arteries, which serve as flow pathways for the hot and cold fluids, thereby enhancing flow distribution and maximizing the heat exchange area. A uniform zinc-based coating was applied to eliminate gas permeability through the ceramic walls. Using a CFD model validated against baseline millichannel experiments (maximum deviation < 5%), they further inferred that the zinc-coated silica exhibits an effective thermal conductivity about 20% higher than the base silica. Experimental results showed that at a hot-side inlet air temperature of 700 °C, the module achieved a volume-based power density of 8.2 MW/m^3^, representing a 71% improvement over a baseline millichannel heat exchanger. Furthermore, this performance was attained with a 22% lower normalized pressure drop penalty (510 Pa/W), confirming excellent energy efficiency. However, further verification is required to assess the long-term durability and mechanical strength of the zinc coating layer under extreme flow velocities and thermal loads.

Conventional polymer heat exchangers are limited in performance due to the low thermal conductivity of polymers and geometric constraints in fabrication. To overcome these challenges, Ahmadi et al. (2022) [62] developed a high-thermal-conductivity polymer heat exchanger featuring a lung-inspired bicontinuous network, fabricated via 3D printing, and experimentally as well as numerically compared its thermal and hydraulic performance in an air–water configuration. Potential leakage issues inherent to 3D-printed polymers were resolved through in-situ epoxy infiltration and post-curing. Using the validated simulations, they further estimated the effective thermal conductivity of the printed polymer structure to interpret the measured performance, highlighting how printing-induced property variations can shift the apparent material advantage. The proposed heat exchanger achieved a thermal effectiveness of 70–80%, with a 101% increase in volume-based power density and a 68% reduction in pressure drop compared with the reference design, demonstrating the superior capability of the lung-inspired architecture. However, the operational range was limited to air–water flows at Re ≈ 10^3^, indicating that further studies on scaling up, alternative working fluids, and broader operating conditions are necessary for practical implementation.

Song et al. (2025) [63] proposed an alveolar biomimetic interlaced hollow lattice metastructure inspired by the interlaced sac-like network of pulmonary alveoli. By nesting cells composed of six variable-diameter hollow pipes, they simultaneously secured a high specific surface area and flow disturbance. By varying the pipe inclination angle, they validated the simulation through thermo-fluid numerical analysis under forced-convection conditions as well as experiments using a fabricated heat exchanger. As a result, this structure, at the unit scale, exhibited a specific surface area that significantly exceeded that of TPMS at the same scale, and in particular, in the high-Re regime, the 45° structure showed enhanced heat transfer, with Nu = 621.44, which is 48.8% higher than that of the BCC. In addition, the 45° structure achieved a PEC of 617.32, nearly twice that of the BCC. The overall heat transfer coefficient was also the best, reaching 1.07 at Re ≈ 32,000. However, the comparisons and validation in this study are based on a specific working fluid and material, as well as the presented operating range and printing-process conditions. In the future, further optimization is required from a reliability perspective, including variations in refrigerant properties, pressure-drop sensitivity during scale-up, and AM defects/dimensional deviations.

To satisfy the requirements of existing aero-engine heat exchangers, it is necessary to overcome the pressure-resistance limitation of plate-fin heat exchangers. Yu et al. (2022) [64] propose a bionic fractal heat-exchanger core inspired by pulmonary alveoli and biological vascular connection structures. They also constructed seven optimized design cases by combining tube wall thickness, tube outer diameter, tube center distance, and chamfer radius, and compared flow and heat-transfer characteristics as well as the heat-exchange area per unit mass and per unit volume. In terms of performance, under numerical-simulation conditions, the heat-exchange rate varied in the range of 217–1104 W. Depending on temperature and flow-rate variations, the efficiency was reported to be as high as 0.77 and as low as 0.56. The CFD results also showed a pronounced hydraulic penalty: the air-side pressure drop increased with inlet temperature and mass flow rate, spanning approximately 147–730 kPa across the tested conditions. While in 3D-printing-based experimental validation, variations of efficiency from 0.767 to 0.558 and heat-exchange rate from 0.837 to 12.31 kW were presented, supporting the numerical trends. This highlights that hydraulic constraints will govern the feasible operating window and motivate correlation-based optimization.

Lung-inspired branched bicontinuous-channel numerical studies have relatively solid computational legitimacy in implementing local mechanisms only when mesh-independence and validation evidence—such as comparisons with experiments and baseline reference models—are explicitly provided. Ahmadi et al. (2022) [62] first conducted prior validation by comparing CFD results with baseline millichannel-module experiments, achieving a maximum deviation of <5%, and then interpreted the performance of a lung-inspired ceramic heat exchanger using those analyses, thereby increasing the credibility of CFD-based conclusions. In addition, Song et al. (2025) [63] presented a mesh-independence procedure, such as selecting the mesh such that the outlet-temperature variation remained below 3%, and reinforced the validity of the numerical results by validating the simulations through separate experiments. Taken together, the CFD results in this section are highly reliable for relative comparisons and for explaining local mechanisms under identical boundary conditions and metrics. However, because of prescribed boundary conditions and as-built geometric deviations, the validation that incorporates actual operating conditions and manufacturing tolerances must be carried out when interpreting absolute performance and scale-up behavior.

Accordingly, lung-inspired heat exchangers increase volumetric heat-transfer performance or adsorption throughput by co-distributing the two fluids throughout the entire porous volume via a bicontinuous, branched network. In particular, a tendency has been reported that the performance gap relative to conventional channels becomes larger at high Re or high flow rates. However, as the structure becomes more complex, such improvements encounter fabrication-reliability limits, because the fabrication basis is largely centered on 3D printing and the experimental validation range is limited. The evidence level is a strength in that numerical analyses and lab-scale experiments have been conducted in parallel, but validation for long-term durability, scale-up, and field reliability is still required. From this perspective, lung-inspired topologies are suitable for situations where flow-distribution uniformity is required, or where performance density under volumetric constraints is a design objective. Conversely, they should be applied conservatively in environments that require coating/bonding reliability.

#### 3.3.4. Network Scale Critical Synthesis and Scale-Up Considerations

TPMS-, leaf-vein-, and lung-network-based designs can all enhance the performance and efficiency of heat exchangers through complex branched or continuous networks. Leaf/branch and lung-network designs improve performance through network-level flow redistribution and secondary-flow generation (and, in some cases, transient/phase-change acceleration), whereas TPMS primarily leverages area amplification and mixing enabled by bicontinuous surfaces. Accordingly, cross-study synthesis should rely on constraint-aware multi-metric evaluation (e.g., PEC/TPF/OPI or Q/ΔP-type indices) rather than a single heat-transfer metric.

However, these structures inherently involve structural risks, such as increased distribution sensitivity or increased pressure-loss penalties. That is, network structures can increase local Nu, but the associated trade-off with increased resistance must be considered. For example, in the study by Zhang et al. (2025) [56], secondary-vein ribs effectively increased the local Nusselt number through boundary-layer disruption and vortex generation, whereas main-vein ribs were reported to potentially reduce PEC because the increase in flow resistance outweighed the heat-transfer enhancement.

In particular, during the scale-up process of heat exchangers, this trade-off between distribution uniformity and increased resistance must be validated. Many studies on network-based heat exchangers are based on lab-scale experiments or numerical analyses conducted under limited lengths, single-fluid conditions, and specific Reynolds-number ranges. This tends to cause insufficient reflection of pressure-drop increases that may appear in real operation, or issues arising from manufacturing tolerances. As the system scale increases, a key challenge is managing flow maldistribution that may be induced by an increased number of branches, extended channel lengths, and accumulated junctions.

Table 5 summarizes the network-scale bioinspired technologies discussed above as a design decision-making framework. The Works/Fails entries synthesize the valid operating window and dominant failure drivers—typically governed by hydraulic constraints, flow maldistribution, manufacturability/tolerance, surface roughness, and durability under scale-up—and do not indicate an absolute superiority or inferiority of any technology. In addition, based on each referenced work, the key evaluation metrics are presented as recommended performance indicators to improve cross-study comparability and real-operating-condition reproducibility.

In summary, network-scale bioinspired heat-exchange technologies are not an area where the focus should be limited to local Nu increases, but rather an area that requires a holistic approach in which flow distribution and pressure loss are designed simultaneously. Future research must go beyond evaluations confined to local metrics such as the Nusselt number or heat-transfer rate, and integrated performance evaluation and validation that include distribution uniformity, accumulated pressure-loss characteristics, and scale-up stability are essential.

## 4. Limitations and Future Perspectives

### 4.1. Current Limitation and Practical Challenges

This review has certain limitations, which stem not only from the constraints of the individual studies reviewed but also from methodological boundaries inherent to the review process. First, many studies have been conducted using small-scale specimens and under single operating conditions. While these studies demonstrate the feasibility of biomimetic applications in heat exchangers, they lack verification of long-term durability and performance under real operating environments. For example, in the surface scale, key issues include wear resistance, chemical stability, lubricant replenishment, and practical performance under environmental conditions. In the network scale, challenges remain regarding structural reliability and stability of optimization under operational stresses. In the texture scale, the high parameter sensitivity of geometric variables makes it difficult to achieve both drag reduction and heat transfer enhancement simultaneously, thereby limiting the generality and applicability of the results.

Second, most studies have been conducted with specific industrial conditions or application purposes in mind. As a result, their findings often exhibit limited generality, and because the experiments were not performed under identical boundary conditions, it remains difficult to directly compare performance outcomes across different studies. In particular, for the studies reviewed under the network scale, the optimization objectives and heat exchanger configurations varied significantly, making it impossible to directly compare their performance results.

Taken together, the limitations identified in this review are not attributable solely to the experimental scale or validation scope of individual studies, but are also closely related to the way performance itself is defined and compared. Although studies conducted under different operating conditions and objective functions each present meaningful results, constraints remain in generalizing them under a common standard or comparing them directly. This suggests that, in future research on biomimetic heat exchangers, there is a need not only for refined designs and validation under real-world conditions, but also for complementary discussions on the perspective itself through which heat-exchange performance is interpreted and evaluated.

### 4.2. Complementary Evaluation Frameworks Beyond Conventional Thermo-Hydraulic Metrics

To date, research on biomimetic heat exchangers has largely advanced around the trade-off between improved heat-transfer performance and increased pressure drop, and heat–fluid-based performance metrics such as PEC have provided an effective criterion for comparing different design concepts. However, because these are transfer-performance-centered metrics defined under specific operating conditions, it may be useful to complement them with a system-level metric such as energy production. Accordingly, drawing on evaluation frameworks from other fields may offer additional insights that complement existing metrics and help guide future design.

In this regard, a series of studies on mammalian nasal cavities interpreted heat and moisture exchange using entropy production as a system-level metric, thereby analyzing exchange efficiency from the perspective of irreversible losses. For example, Magnanelli et al. (2016) [65] constructed a dynamic model that reflects the geometric morphology of the reindeer nasal cavity and quantified geometric effects by comparison with a simple cylindrical structure having the same total volume and contact area. This study suggests that the complex nasal-cavity structure of mammals goes beyond merely increasing surface area, and can distribute entropy production more uniformly.

Studies on marine mammals likewise provide a similar perspective. Cheon et al. (2023) [66] extracted the cross-sectional area of the flow path and the perimeter of the air–tissue interface from CT-based nasal-cavity geometries of *Erignathus barbatus* and *Monachus monachus*, and applied them to a quasi-1D dynamic model based on mass and energy balances. The analysis showed that, under identical environmental conditions, entropy production decreases significantly as the perimeter of the maxilloturbinate increases, and they discussed that, in terms of energy dissipation, the Arctic species is relatively advantageous due to geometric differences. Subsequently, Flekkøy et al. (2023) [67] demonstrated, through a thermo-hydrodynamic model, that ice formation must be considered to reproduce expiratory temperatures in low-temperature environments. Furthermore, Cheon et al. (2024) [68] evaluated entropy production under multiple environmental temperatures, relative humidities, and physiological conditions using a CFD model for the maxilloturbinate structure of seals.

Although these studies are not cases directly implemented as engineering heat exchangers, they are meaningful as conceptual reference cases showing that minimizing irreversible losses can be considered a design objective in the process of evaluating heat-exchange performance. This evaluation framework, rather than replacing existing heat–fluid performance metrics, provides applicability as a complementary perspective for future biomimetic heat-exchanger design and analysis.

The biological systems mentioned in this section are not presented as direct design templates for heat exchangers, but are introduced as conceptual reference cases to present an evaluation framework and interpretive perspective that can supplement existing heat–fluid performance metrics.

Taken together, future research should prioritize the scaling-up of biomimetic designs and the experimental validation of real-world performance to effectively enhance heat exchanger efficiency through nature-inspired strategies. In particular, further investigation is needed in areas such as operating condition optimization, techno-economic feasibility in fabrication and maintenance, material compatibility and long-term durability, and the scalability of biomimetic designs for use in general-purpose industrial heat exchangers. In this process, supplementing existing performance-evaluation metrics using indicators such as entropy production is expected to help refine comparisons among design alternatives and the specification of design targets.

## 5. Conclusions

As demonstrated throughout this review, biomimetic design has emerged as a promising and innovative approach for enhancing the performance and reliability of modern heat exchangers. These systems inherently face challenges such as frost formation, fouling, and pressure drop, yet bioinspired technologies provide effective solutions across multiple scales, offering tailored strategies to mitigate these fundamental limitations.

For instance, applying superhydrophobic properties inspired by lotus leaves or slippery surface characteristics modeled after Nepenthes pitcher plants to heat exchanger surfaces can enable self-cleaning functionality, effectively preventing fouling. In particular, for low-temperature heat exchangers, such surface modifications can suppress frost formation, mitigating potential efficiency losses. Similarly, by replicating the scale textures of fish or the micro-patterns of various plant and animal surfaces, the frictional resistance between the fluid and the wall can be minimized, thereby reducing pressure drop without compromising heat transfer performance—an improvement that can be quantitatively evaluated using the PEC.

At the macroscopic level, depending on industrial requirements, overall heat exchanger geometry and flow channel arrangements can also be optimized to improve thermal performance. Such designs draw inspiration from branching vein structures in leaves and trees, TPMS, and the gas exchange mechanism of the human lung. As illustrated by these recent studies, biomimetic technologies hold significant promise for achieving advanced performance enhancement and energy efficiency optimization in next-generation heat exchangers.

Recent research trends clearly demonstrate the broad applicability and potential of bioinspired technologies across multiple scales for improving the energy efficiency of heat exchangers. Once commercial-scale demonstrations generate sufficient operational data, these technologies are expected to drive transformative advancements in thermal energy management within modern industry.

## Figures and Tables

**Table 1:** Surface-scale bioinspired technologies: operating window, key metrics, and implementation risks.

Technology	Objective/Mechanism	Valid Operating Window (Works/Fails)	Key Evaluation Metrics	Implementation Risks	Ref.
Superhydrophobic Surface	Suppress droplet spreading to promote roll-off, droplet jumping, and surface refreshment, thereby maintaining stable dropwise condensation.	**Works:** Operating regimes where frosting and defrosting burdens directly translate into efficiency penalties. **Fails:** When micro/nanostructures progressively degrade due to wear and contamination, leading to Cassie-state failure (Cassie–Wenzel transition).	Sliding angle; contact-angle hysteresis (CAH); critical departure diameter/period	– Wear and contamination accumulation – Potential thermal-resistance trade-off due to added coating/polymer layers or trapped air layers	[1,5,6,7,8,12]
SLIPS	Introduce a lubricant layer to achieve ultra-low hysteresis, enabling enhanced droplet mobility and reduced adhesion (condensate/ice).	**Works:** Humid, low-temperature environments where anti-icing/drainage performance is critical. **Fails:** When lubricant depletion is accelerated and/or under high-contamination conditions that promote lubricant loss or surface damage.	CAH; ice-adhesion strength; frost delay time; lubricant depletion rate	– Need for lubricant replenishment and re-application – Lubricant loss/depletion during long-term operation	[9,12,13,14,15]
Self-replenishing SLIPS	Achieve long-term stability by continuous lubricant resupply, sustaining a slippery interface over extended cycles.	**Works:** Long-cycle operation where maintenance access is limited and lubricant stability must be maintained. **Fails:** When performance is highly sensitive to operating conditions outside the designed window, or when the resupply mechanism is insufficient under real contamination/flow conditions.	Performance retention time; replenishment stability	– Potential pinning and flow-pattern alteration, with increased structural complexity – Contamination management and maintenance requirements	[11]

**Table 3:** Thermo-hydraulic performance reported as separated heat-transfer gain and hydraulic penalty/benefit.

Biomimetic Target	Heat Transfer Gain (vs. Baseline)	Hydraulic Penalty/Benefit (vs. Baseline)	Validity/Trade-Off	Ref.
Fish-scale	Nu +38.2%	f +150.2%	High Nu gain, high f penalty; narrow optimum	[26]
Fish-scale	Type 1: Nu +12.3–25.2%; Type 2: Nu +14.9–38.0%	Type 2: f/f_0_ up to 1.50	Re-limited benefit; type-dependent trade-off	[28]
Fish-scale	Nu +14%	f −5%	Best case improves both; larger I_*f*_/D_*h*_ increases Nu and f	[29]
Crab shell	j factor +22.57%	f +26.11%	j gain with f rise; parameter-sensitive	[30]
Shark Denticle	Nu +13.1%	ΔP average : 19% ; peak : 26%	Comparator-dependent; not universally best	[31]

**Table 4:** Texture-scale bioinspired technologies: operating window, key metrics, and implementation risks.

Technology	Objective/Mechanism	Valid Operating Window (Works/Fails)	Key Evaluation Metrics	Implementation Risks	Ref.
Drag-reduction surfaces	Suppress near-wall turbulence structures to reduce skin friction. Riblets (streamwise grooves) constrain spanwise motions and vortex interactions, thereby reducing C_*f*_. Some patterns can induce secondary flows, potentially affecting heat-transfer-related behavior as well.	Mainly turbulent boundary layers/turbulent channel flows. Key scaling is often not Re itself but wall-unit scaling, where an optimum emerges.	Drag reduction, C_*f*_ reduction ratio, wall shear stress, friction factor, dimensionless geometric parameters such as s^+^/ Ag^+^	– High sensitivity to alignment (yaw/misalignment). – Strong dependence on tolerances, wear, contamination/fouling. – Frost/scale/particle deposition can become a critical failure mode for heat-exchanger.	[16,17,18,19,20,24,39,43]
Compact HX textures (channels, fins, tubes)	Surface/shape textures targeting simultaneous improvement of heat transfer and reduction of Δp. Examples: denticle/streamlined fins, fish-scale protrusions on tubes; control mixing, secondary flows, and boundary- layer separation.	Internal flows (tubes/fin-channels), typically turbulent to transitional regimes. (e.g., PFHE denticle fins, fish-scale tubes).	Nu, h, Δp, f, PEC/TPF	– Nonlinear trade-off. – Manufacturing difficulty and reproducibility. – Cleanability and susceptibility to fouling.	[26,28,30,31]
Microchannels/heat sinks	Enhance mixing via micro-ribs/fins/asymmetric distributions, or improve heat transfer and exergy efficiency using nanofluids and/or magnetic fields.	Typically microchannel forced convection at Re = *O*(10^2^–10^3^) (e.g., Re ≈ 700–1400). Comparisons often performed under fixed heat-flux boundary conditions.	Nu, friction factor, Δp, PEC, temperature reduction rate, thermal resistance	– Clogging/particle-related issues. – Increased pumping power demand. – Under frosting/icing assumptions, micro-passages face a high risk of ice-plug formation.	[27,29,32,40,42]
Patterned plate heat exchangers and materials	Use lung/gill/trachea-type patterns to co-design surface area, flow distribution, and secondary flows. Complex patterns enabled by additive-manufacturing (AM), while material choice (e.g., steel/Al/Ti, PA12) directly affects thermo-hydraulic performance and durability.	Mainly water-based, single-phase heat exchange. Often evaluated by jointly comparing ε – Δp – pumping power across flow- rate variations.	Heat-transfer rate Q, overall U, effectiveness ε, Δp, pumping power, COP, PEC	– AM-related surface roughness/internal defects/leakage risk. – Cleanability challenges for complex patterns. – Material-related risks (strength, creep, temperature/chemical compatibility, etc.)	[33,34,35,36,37,38]

**Table 5:** Network-scale bioinspired technologies: operating window, key metrics, and implementation risks.

Technology	Objective/Mechanism	Valid Operating Window (Works/Fails)	Key Evaluation Metrics	Implementation Risks	Ref.
TPMS core	Leverage the high surface-area-to-volume ratio and 3D continuous flow pathways of TPMS architectures to promote mixing and vortex generation.	**Works:** Regimes where heat-transfer enhancement is prioritized under fixed pumping-power and/or volumetric constraints. **Fails:** Systems with a tight pressure-drop budget, or when manufacturing-induced surface roughness and wall-thickness increases cannot be controlled; performance may also become condition-dependent at low flow rates/low Re.	Nu, U, Q, ΔP, f, PEC (typically compared under an identical pumping-power basis)	– AM defects, surface roughness, and increased wall thickness can distort interpretation of absolute performance. – As-built deviations can undermine simulation–experiment agreement and scale-up reliability.	[44,45,48,49,50]
Functionally graded TPMS	Optimize the heat-transfer vs. pressure-drop trade-off via functional gradation (e.g., cell-size variation, level-set modulation, and filtering) to improve flow uniformity and thermo-hydraulic performance.	**Works:** Applications where the target Reynolds-number range is relatively well-defined, enabling grading to be tuned to a known operating window. **Fails:** When the optimum is non-monotonic or highly condition-dependent, or when performance deteriorates outside the tuned window.	Nu, U, Q, ΔP/f, PEC	– High implementation difficulty of multi-parameter gradation. – Increased risk of design manufacturing mismatch due to many optimization variables and sensitivity to as-built tolerances.	[47,51,52]
TPMS infill/tube- filling enhancement	Fill cylindrical tubes with TPMS lattices to increase turbulence intensity, mixing, and heat-transfer augmentation within an existing tube-based framework.	**Works:** Retrofittable conventional tube-based systems where internal enhancement is preferred over a complete redesign. **Fails:** When relative density is high and/or the structure is sheet-like, which can impose excessive hydraulic penalties.	Composite indices such as (Q/Q_0_)/(ΔP/ΔP_0_), along with turbulence intensity and ΔP.	– Outcomes are strongly governed by relative density, printing defects, and material selection/thermal conductivity. – As-built imperfections can dominate both ΔP and heat-transfer trends.	[46]
Leaf/branch-inspired flow distribution	Use branched and hierarchical network architectures (leaf veins/tree branches) to uniformly distribute flow or optimize diffusion pathways.	**Works:** Systems where distribution uniformity is performance-critical, and where terminal-branch maldistribution dominates overall performance. **Fails:** When manufacturing variability, assembly/installation errors, or non-uniform surface roughness induce severe maldistribution.	ΔP–Q correlation, Nu–Re correlation, terminal-channel deviation (flow-distribution metric), TPF	– Highly sensitive to manufacturing and assembly tolerances. – Need to ensure structural robustness/ mechanical integrity.	[55,57,58,59]
Leaf/branch-inspired rib and channel augmentation	Introduce rib networks to disturb boundary layers and induce secondary flows/vortices, enhancing convective heat transfer.	**Works:** Microchannel-dominated regimes where Nu is the primary bottleneck, and rib optimization enables PEC > 1. **Fails:** When the pressure-drop penalty offsets the heat-transfer gain.	Nu, ΔP, PEC, sensitivity to rib height and diameter ratio	– ΔP penalty and potential flow non-uniformity. – Performance can drop sharply outside the optimized parameter range. – Requires re-validation under refrigerant changes and scale-up.	[56]
Leaf/branch-inspired thermal diffusion and phase-change enhancement	Optimize heat-spreading pathways in solids or PCMs using leaf/branch-like fins or lattice networks, thereby accelerating internal heat diffusion and phase- change dynamics.	**Works:** PCM-based systems where transient heat spreading is the dominant limitation. **Fails:** Regimes dominated by forced-convection heat exchange, or cases with large interfacial contact resistance (which can suppress the benefit of internal heat- spreading networks).	Solidification time, average and peak temperature, thermal response time	– PCM leakage and cycle durability issues. – Sensitivity to 3D-printing tolerances and post-processing. – Thermal-resistance redistribution during scale-up (non-uniform effective conduction paths).	[53,54]
Lung-inspired bicontinuous network	Mimic bronchial–alveolar principles to build a branched bicontinuous/interwoven network that co-distributes two fluids across the full volume, increasing volumetric performance density and/or adsorption throughput.	**Works:** Systems targeting high volumetric performance density and uniform distribution (e.g., high-Re/high-flow regimes, adsorption-based DCHX). **Fails:** When scale-up causes accumulated ΔP to become prohibitive, or when fabrication cannot ensure reliability of complex 3D-printed structures.	Volumetric power density, ΔP, Nu, PEC	– 3D-printing defects and dimensional deviations. – Need for validation of coatings/infiltration/sealing and leakage reliability. – Limited evidence on long-term durability and field validation.	[60,61,62,63,64]

## Data Availability

No new data were created or analyzed in this study.

## Abbreviations

The following abbreviations are used in this manuscript:

PEC	Performance Evaluation Criterion
PHE	Plate Heat Exchangers
PCHE	Printed Circuit Heat Exchangers
MCHE	Micro Channel Heat Exchangers
PCM	Phase Change Materials
CFD	Computational Fluid Dynamics
SEM	Scanning Electron Microscope
ASHP	Air-Source Heat Pump
PDMS	Polydimethylsiloxane
SLIPS	Slippery Liquid-Infused Porous Surfaces
CAH	Contact-Angle Hysteresis
LES	Large-Eddy Simulation
DNS	Direct Numerical Simulation
NACA	National Advisory Committee for Aeronautics
CPHE	Compact Plate Heat Exchanger
COP	Coefficient Of Performance
AM	Additive Manufacturing
TPMS	Triply Periodic Minimal Surface
TPF	Thermal Performance Factor
